# Two intracellular and cell type-specific bacterial symbionts in the placozoan *Trichoplax* H2

**DOI:** 10.1038/s41564-019-0475-9

**Published:** 2019-06-10

**Authors:** Harald R. Gruber-Vodicka, Nikolaus Leisch, Manuel Kleiner, Tjorven Hinzke, Manuel Liebeke, Margaret McFall-Ngai, Michael G. Hadfield, Nicole Dubilier

**Affiliations:** 1 Max Planck Institute for Marine Microbiology, Bremen, Germany; 2 Department of Plant and Microbial Biology, North Carolina State University, Raleigh, NC USA; 3 Department of Pharmaceutical Biotechnology, Institute of Pharmacy, University of Greifswald, Greifswald, Germany; 4 Institute of Marine Biotechnology, Greifswald, Germany; 5 Department of Geoscience, University of Calgary, Calgary, Alberta Canada; 6 Kewalo Marine Laboratory, Pacific Biosciences Research Center, University of Hawai’i at Mānoa, Honolulu, HI USA

**Keywords:** Symbiosis, Water microbiology

## Abstract

Placozoa is an enigmatic phylum of simple, microscopic, marine metazoans^1,2^. Although intracellular bacteria have been found in all members of this phylum, almost nothing is known about their identity, location and interactions with their host^3–6^. We used metagenomic and metatranscriptomic sequencing of single host individuals, plus metaproteomic and imaging analyses, to show that the placozoan *Trichoplax sp.* H2 lives in symbiosis with two intracellular bacteria. One symbiont forms an undescribed genus in the Midichloriaceae (Rickettsiales)^7,8^ and has a genomic repertoire similar to that of rickettsial parasites^9,10^, but does not seem to express key genes for energy parasitism. Correlative image analyses and three-dimensional electron tomography revealed that this symbiont resides in the rough endoplasmic reticulum of its host’s internal fibre cells. The second symbiont belongs to the Margulisbacteria, a phylum without cultured representatives and not known to form intracellular associations^11–13^. This symbiont lives in the ventral epithelial cells of *Trichoplax*, probably metabolizes algal lipids digested by its host and has the capacity to supplement the placozoan’s nutrition. Our study shows that one of the simplest animals has evolved highly specific and intimate associations with symbiotic, intracellular bacteria and highlights that symbioses can provide access to otherwise elusive microbial dark matter.

## Abstract (web summary)

Using a multi-omics approach, together with imaging analyses, the authors characterize the two intracellular bacterial symbionts of *Trichoplax*, one of the simplest animals.

## Main

Placozoa are marine invertebrates at the base of the animal tree and are considered among the simplest animals known. These millimetre-sized benthic animals can be easily cultured and are considered key models for understanding metazoan evolution, developmental biology and tissue formation^1,14–16^. Electron microscopy studies as early as in the 1970s revealed the presence of intracellular bacteria in these animals^3–6^. Remarkably, nearly five decades later, only very little is known about the biology of these symbionts and their interactions with their hosts.

The phylum Placozoa encompasses 19 cryptic species, on the basis of mitochondrial haplotypes^2,6^. These benthic animals do not have a mouth or gut and feed on algae and bacterial biofilms by external digestion and subsequent uptake via their ventral epithelium^17,18^. All placozoans have three cell layers and six morphologically differentiated cell types^3,6,19,20^. The thick ventral epidermis consists of ciliated epithelial cells in which glandular and lipophilic cells are irregularly interspersed^17,18^. Ciliated epithelial cells make up the thin dorsal epidermis in which crystal cells occasionally occur. An internal meshwork of fibre cells, sandwiched between the two epidermal layers, connects the ventral and dorsal body walls^20^. Intracellular symbionts were first described in these fibre cells^3,5,20^. The bacteria were present in all seven haplotypes examined, independent of sampling site or time, and were hypothesized to reside in the lumen of the rough endoplasmic reticulum (rER)^3,5,6,20^. Persistent and stable residence of a bacterium in the rER of a host would be remarkable as the vast majority of intracellular symbionts live in the cytoplasm or vacuoles, and the few known exceptions inhabit the nucleus or mitochondria^21–23^.

In this study, we focused on the *Trichoplax sp.* haplotype H2 (*Trichoplax* H2), previously reported to host two bacterial morphotypes^5^. Sequencing of placozoan genomes consistently yielded rickettsial and other bacterial sequences^6,24,25^. However, as thousands of host individuals were pooled for these analyses, it was neither clear whether these bacterial sequences originated from contaminants or symbionts nor whether they were present in all host individuals. Our recent advances in high-throughput sequencing of single placozoan individuals, together with correlative imaging analyses and three-dimensional (3D) reconstruction, allowed us to explore the patterns, structure and function of the placozoan symbiosis at the individual and cellular level.

The *Trichoplax* H2 microbiome is dominated by two bacterial symbionts. We isolated a placozoan H2 haplotype lineage from a seawater tank at the Kewalo Marine Laboratory, University of Hawai’i (Supplementary Fig. 1). To characterize the microbiome of this *Trichoplax* H2, we combined highly sensitive DNA and RNA extraction and library preparation protocols to sequence the metagenomes and metatranscriptomes of microscopic single individuals that have an estimated biovolume of 0.02 µl and from which we could isolate 0.5 to 4 ng of nucleic acids (*n* = 5). All five individuals had similar microbial communities based on 16S ribosomal RNA (rRNA) gene reads, but only two taxa were consistently dominant in all five host individuals (Supplementary Fig. 2 and Supplementary Table 1).

**Fig. 1:**
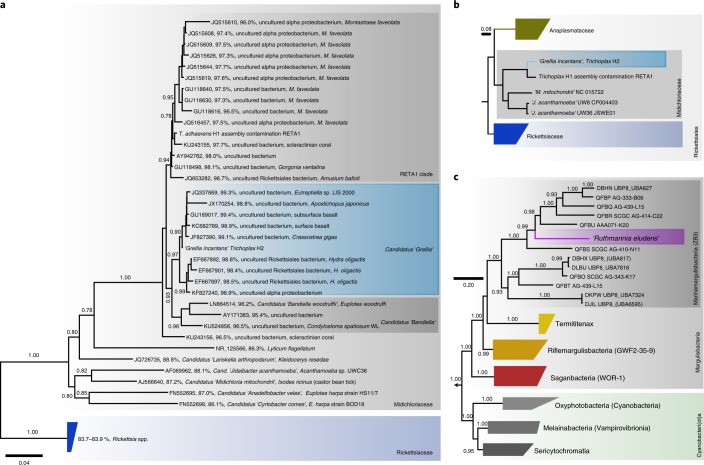
Phylogenetic analyses of the *Trichoplax* H2 symbionts. ‘*G. incantans*’ represents an undescribed genus in the Midichloriaceae (Rickettsiales) and ‘*R. eludens*’ is a Marinamargulisbacterium (Margulisbacteria). Bootstrap support values below 0.5 are not shown. Scale bars indicate substitutions per site. Colours and shades of grey indicate taxonomic groups. **a**, A 16S rRNA tree of *‘G. incantans’* and related Midichloriaceae. For each sequence, the accession number, the percentage identity to ‘*G. incantans*’ and the published taxonomic names and hosts (where available) are indicated. **b**,**c**, Phylogenomic analyses using 43 conserved marker genes based on metagenome-assembled genomes and reference genomes: ‘*G. incantans*’ and Midichloriaceae are placed in the Rickettsiales (**b**) and ‘*R. eludens*’ in the Margulisbacteria (**c**). Phylum-level classification follows the Genome Taxonomy Database. Taxon names from the Genome Taxonomy Database are indicated in parentheses where available.

The first and most abundant 16S rRNA phylotype was an alphaproteobacterium from the family Midichloriaceae (Rickettsiales)^7^ (Fig. 1a). Midichloriaceae are obligate intracellular, often pathogenic, bacteria found in protists and animals, including humans^8^. In 16S rRNA analyses, the *Trichoplax* H2 midichloriacean phylotype formed an unnamed lineage that consisted of sequences recovered from diverse invertebrate hosts and sequences from subsurface sediment samples (98.4–99.4% pairwise identity; Fig. 1a). We recovered a high-quality^26^ 1.26 Mb metagenome-assembled genome that included the midichloriacean 16S rRNA phylotype. Sequences from the *Trichoplax adhaerens* haplotype H1 genome project^15^ included a midichloriacean 16S rRNA gene fragment and a partial genome of a rickettsial phylotype (RETA1) was also recovered^25^. Phylogenetic analyses based on the 16S rRNA gene and phylogenomic analyses based on 43 conserved marker genes placed the *Trichoplax* H2 phylotype and *Trichoplax* H1 RETA1 in the Midichloriaceae. The *Trichoplax* H2 and H1 phylotypes were phylogenetically distinct and, according to amino acid sequence identity, these two symbionts belong to two separate but undescribed genera, with *Candidatus* ‘*Bandiella*’^27,28^ as the closest characterized genus (Fig. 1a,b, Supplementary Note 1). We propose the *Candidatus* taxon ‘*Grellia incantans*’ for the midichloriacean phylotype from our haplotype H2 isolate (see Supplementary Note 1 for description and etymology).

The second most abundant and consistently present bacterial taxon in the *Trichoplax* H2 metagenomes belonged to the Margulisbacteria, a phylum without isolated representatives that forms the sister clade to Cyanobacteriota^11–13,29^. No 16S rRNA gene sequences with >90% identity to this bacterium were found in public sequence databases, warranting the establishment of a taxon at the genus or even family level. We therefore propose the *Candidatus* taxon ‘*Ruthmannia eludens*’ for this bacterium (see Supplementary Note 2 for a detailed description and etymology). Using metagenomics binning, we recovered a high-quality 1.51 Mb metagenome-assembled genome for ‘*R. eludens*’. Our phylogenomic analyses confirmed our 16S rRNA gene analysis and placed ‘*R. eludens*’ in the Marinamargulisbacteria (Margulisbacteria) (Fig. 1c). Marinamargulisbacteria are aquatic bacteria that occur worldwide^11,13^. ‘*R. eludens*’ is distantly related to single-cell amplified genomes and metagenome-assembled genomes from marine pelagic samples^13^ (Fig. 1c). Marinamargulisbacteria are known only from sequence-based studies, with recovered draft genomes of 0.5–2.0 Mb, and all genomes are classified as medium to low quality^26^. Despite the small genome size, our metagenome-assembled genome was classified as a high-quality draft genome (Supplementary Note 2).

**Fig. 2:**
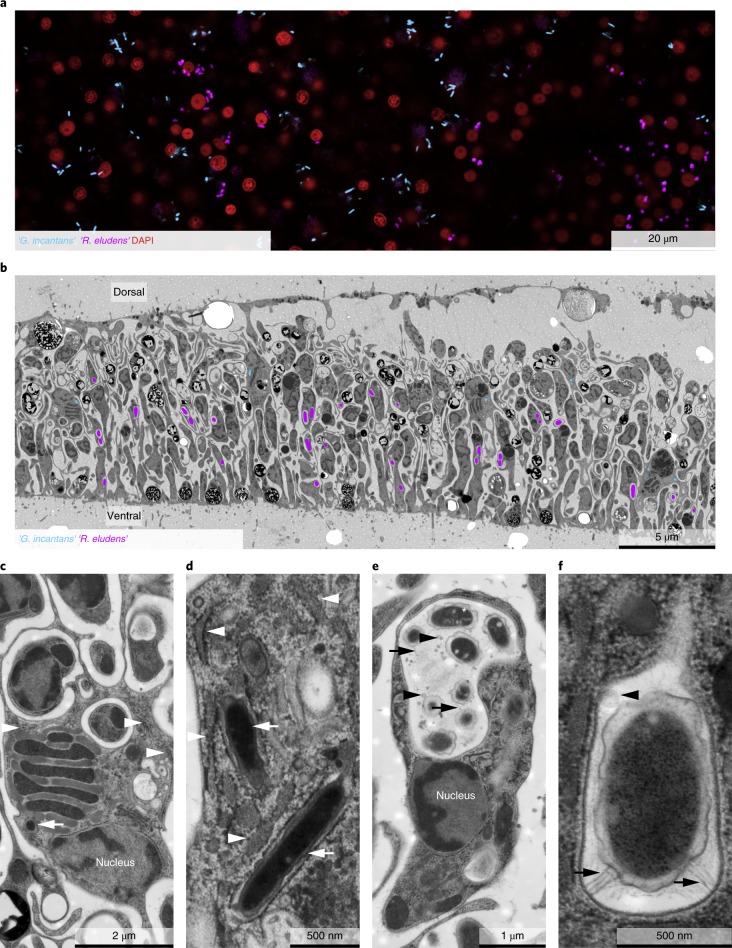
‘*R. eludens*’ and ‘*G. incantans*’ are specific to two spatially segregated host cell types. **a**, A false-coloured FISH image using probes specific for ‘*G. incantans*’ (GRIN-61-2, Atto-647) and ‘*R. eludens*’ (RUEL-846-22, Atto 594); host nuclei are stained with 4,6-diamidino-2-phenylindole (DAPI). The results are representative of five independent experiments. **b**, A TEM image of a cross-section of *Trichoplax* H2 with ‘*G. incantans*’ and ‘*R. eludens*’ indicated in false colour (for raw image data see Supplementary Fig. 5). **c**,**d**, TEM images of fibre cells. ‘*G. incantans*’ is indicated with white arrows and the rER with white arrowheads. **e**,**f**, TEM images of ventral epithelial cells containing ‘*R. eludens*’. Outer membrane vesicles are indicated with black arrowheads, fimbriae-like structures with black arrows and internal structures by a white asterisk. Results in **b**−**f** are representative of three independent experiments.

Both symbionts are intracellular, spatially segregated and specific to the host cell type. We used fluorescence in situ hybridization (FISH; Fig. 2a; Supplementary Table 2) to link the bacterial sequences to their morphotypes and visualize the distribution of the two symbionts in *Trichoplax*. No bacteria except the two symbionts ‘*G. incantans*’ and ‘*R. eludens*’ were detected in all the placozoan individuals examined (Fig. 2a; Supplementary Fig. 4). ‘*G. incantans*’ was thin and rod-shaped (≤1.2 × 0.30 μm^2^; Fig. 2a and Supplementary Fig. 4). In contrast, ‘*R. eludens*’ had a wider and stouter rod-shaped morphology (≤1.2 × 0.47 μm^2^; Fig. 2a and Supplementary Fig. 4; for details see Supplementary Notes 1 and 2). Our correlative FISH and transmission electron microscopy (TEM) analyses of five *Trichoplax* H2 individuals revealed that the two bacterial symbionts were always intracellular, spatially segregated and specific to one of the six host cell types (Fig. 2b and Supplementary Figs. 5−7). ‘*G. incantans*’ was observed only in fibre cells and was the only bacterium located in these cells (Fig. 2b and Supplementary Figs. 5 and 6). All ‘*G. incantans*’ cells were surrounded by a host membrane that was densely covered with ribosomes (Fig. 2c,d and Supplementary Fig. 6; *n* = 49 symbiont cells in 9 specimens). Similar host structures surrounding the bacteria in other *Trichoplax* lineages were interpreted as indicating that the bacteria reside inside the host’s rER^3^. An alternative interpretation for such host membrane structures was shown for the human intracellular pathogens *Brucella* and *Legionella*, as well as the amoebal midichloriacean parasite *Candidatus* ‘*Jidaibacter*’. These bacteria remodel the phagosome surfaces of their hosts so that they become covered by host ribosomes as an effective strategy for avoiding digestion by their hosts^21,30^.

**Fig. 3:**
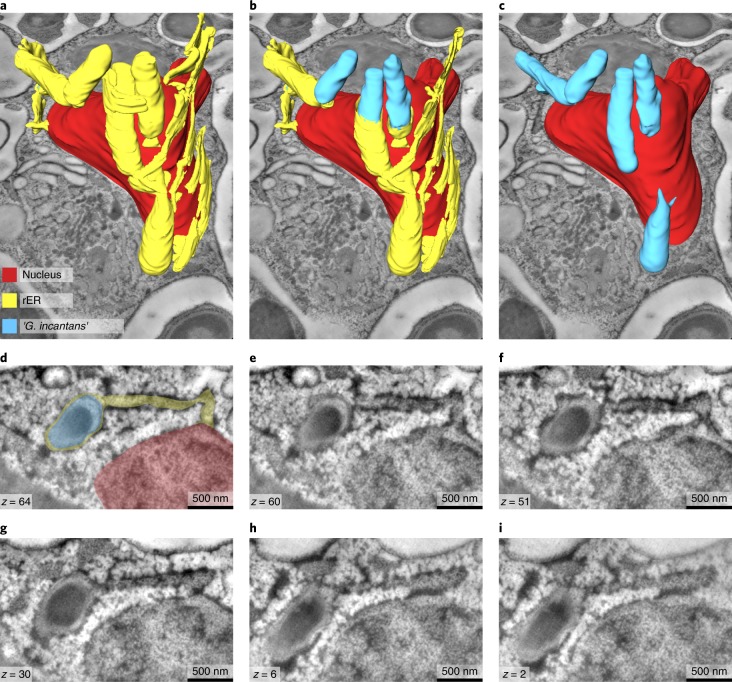
‘*G. incantans*’ lives in the rER of *Trichoplax* H2. All panels are the results from a single experiment. **a**−**c**, 3D volume rendering of reconstructed ‘*G. incantans*’, the rER and the nucleus of a fibre cell, superimposed on a virtual slice of the 3D TEM tomography stack; the rER has been virtually removed to partially (**b**) and fully (**c**) show the symbionts within the rER lumen. No scale bar is shown as the scale varies with perspective. **d**−**i**, Selected tomography slices (on which the 3D reconstruction is based) at various depths (see *z* values indicated) show the connection between the nucleus, the rER and the bacteria. For ease of interpretation, **d** has been false-coloured using the colour key from **a**. For raw data, see Supplementary Fig. 8.

To resolve the subcellular architecture of ‘*G. incantans*’ symbiosis, we used high-resolution 3D TEM tomography to determine whether the structures surrounding the symbiont cells were remodelled phagosomes or rER. Our 3D electron tomographic reconstructions revealed that the ribosome-covered membranes, in which ‘*G. incantans*’ occurred, formed networks that were connected to the nuclear envelope^31^. This indicates that the structure in which ‘*G. incantans*’ is embedded is in fact rER. ‘*G. incantans*’ symbionts were only observed in the rER, some even within the same rER lumen, and never in other host structures (Fig. 3; Supplementary Fig. 8; Supplementary Video 1). These analyses suggest that ‘*G. incantans*’ persistently resides in the rER of its host. The second symbiont, ‘*R. eludens*’, colonized only the ventral epithelial cells. These symbionts were always found within cytoplasmic vacuoles of the host (Fig. 2e,f). The vacuoles contained numerous membrane-bound vesicles, presumably outer membrane vesicles produced by ‘*R. eludens*’ (Supplementary Fig. 7). Thin, tubular structures that resemble fimbriae appeared to connect the bacterial cells to the host vacuole membrane (Fig. 2f; Supplementary Fig. 7).

Bacteria that live inside animal cells are known from only 6 of the 114 recognized bacterial phyla^32^. The number of bacterial phyla with representatives that can live as intracellular symbionts has not increased since the characterization of Mycoplasmatales in the early 1960s, despite huge advances in the sequencing of animals from a wide range of phyla and environments that have led to the discovery of numerous lineages of microbiota^11,32^. Marinamargulisbacteria is one of the most phylogenetically remote clades of bacteria, discovered through high-throughput sequencing of environmental samples^33^. The remote position of the placozoans in the animal tree of life has probably contributed to this late discovery of Margulisbacteria as the seventh bacterial phylum with intracellular symbionts of animals. Our study of the *Trichoplax* microbiome highlights how bacteria captured by eukaryotes provide a route for studying bacterial groups that are otherwise known only from sequences found in water or sediment samples.

**Fig. 4:**
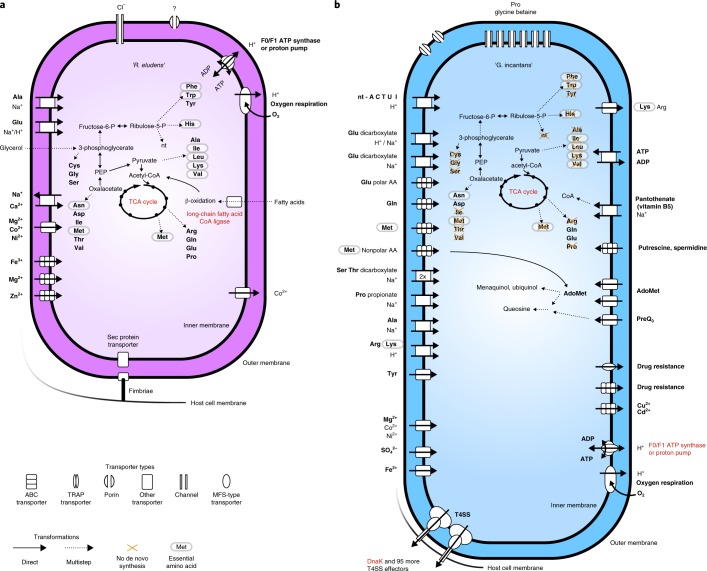
‘*R. eludens*’ has versatile biosynthesis pathways, whereas ‘*G. incantans*’ depends on the import of most nutrients from its host. Physiological reconstructions based on RAST annotations and Pathway Tools metabolic modelling. Functions that are discussed in the text and highly expressed are indicated in red. **a**, ‘*R. eludens*’. **b**, ‘*G. incantans*’. Bold font indicates primary function. ABC, ATP-binding cassette; AdoMet, *S*-adenosyl-l-methionine; MFS, major facilitator superfamily; nt, nucleotide; nt-ACTUI, the nucleotides a cell can import (all but guanine); P, phosphate; PEP, phosphoenolpyruvate; T4SS, type IV secretion system; TRAP, tripartite ATP-independent periplasmic.

‘*R. eludens*’ gains nutrition from lipids degraded by its host. We sequenced the metatranscriptomes of the same single placozoan individuals that were used for metagenomic analyses (*n* = 3) and generated metaproteomes from pooled samples of 10 to 30 individuals (*n* = 3) to investigate the physiology of ‘*R. eludens*’. Physiological modelling of these expression data revealed that ‘*R. eludens*’ is an aerobic chemoorganoheterotroph, with a complete tricarboxylic acid (TCA) cycle that generates energy and biomass from glycerol and the *β*-oxidation of fatty acids (Fig. 4a; Supplementary Table 3). The source of the glycerol and fatty acids is probably lipids from the algal diet of the host. Our analyses of the host’s transcriptome revealed that *Trichoplax* H2 expressed several lipases, most probably for the digestion of the algae it feeds on (Supplementary Table 4). These host lipases hydrolyze lipids to glycerol and fatty acids. The genome of ‘*R. eludens*’ also encodes lipases that would allow ‘*R. eludens*’ to digest lipids independently of its host. Interestingly, we found neither transcripts nor peptides for these symbiont lipases, suggesting that ‘*R. eludens*’ relies on the lipases expressed by its host (Supplementary Table 3).

The transfer of glycerol and even-chain fatty acids from the host to ‘*R. eludens*’ probably occurs passively, as they can easily diffuse through cell membranes. We predict that the fatty acids are taken up and activated by ‘*R. eludens*’ on the basis of its high expression of a long-chain fatty acid coenzyme A (CoA) ligase (among the top 25% of expressed genes; Fig. 4a; Supplementary Table 3). The fatty acids are then probably catabolized to acetyl-CoA and respired, as indicated by the expression of all the genes needed for *β*-oxidation and the oxidative TCA cycle. However, the anabolic incorporation of fatty acids is unlikely, as we could not detect the genes for the glyoxylate shunt.

‘*R. eludens*’ encoded genes for synthesizing all nucleotides and amino acids, including the nine amino acids considered essential for animals. However, we found no genomic or transcriptomic indications that ‘*R. eludens*’ exports nutrients to its host, for example via amino acid exporters (see Fig. 4a and Supplementary Note 3). Moreover, in our TEM analyses, we found no evidence for the intracellular, lysosomal digestion of ‘*R. eludens*’, such as lamellar bodies or tertiary lysosomes commonly observed in other nutritional symbioses^34,35^. Our ultrastructural analyses did, however, reveal large numbers of putative outer membrane vesicles in the host vacuole surrounding ‘*R. eludens*’ (Fig. 2e,f and Supplementary Fig. 7). It is tempting to speculate that the host takes up outer membrane vesicles produced by ‘*R. eludens*’ via phagocytosis and thus supplements its diet, as the host lacks synthesis pathways for essential amino acids. However, the beneficial effects of such putative amino acid provisioning by ‘*R. eludens*’ are not clear, given that the animal’s algal diet may contain sufficient amounts of essential amino acids.

‘*G. incantans*’ has the genes for energy parasitism but does not express them: it lives in the rER of fibre cells and seems to be a typical Rickettsiales based on genomic features alone, namely a heterotroph that relies on its host for biomass and energy generation (Fig. 4b). The ‘*G. incantans*’ genome encodes the hallmark feature for intracellular energy parasites that is present in all Rickettsiales genomes: a fully functional ADP/ATP-translocase for importing ATP from its host^9^. In contrast to all other known energy parasites, we found no transcripts or respective peptides of the ADP/ATP-translocase in ‘*G. incantans*’ (Supplementary Table 5). Instead, ‘*G. incantans*’ generated ATP with an ATP synthase, and the subunits a and b were highly expressed in the bacterium’s proteome (Supplementary Table 6). Compared to the typical energy-parasitic lifestyle of cytosolic Rickettsiales that rely on ATP imported from their hosts^10^, the ability of ‘*G. incantans*’ to synthesize ATP by itself likely lowers its detrimental impact on its host considerably.

High transcription of key genes of the oxidative TCA cycle and the presence of a complete electron transport chain in the genome, with some of the subunits of the electron transport chain among the most highly transcribed genes, suggests that the proton gradient for ATP synthesis is fuelled by oxidative phosphorylation (Fig. 4b and Supplementary Table 5). An incomplete glycolysis pathway and several importers for *α*-ketoacids and C4-dicarboxylates suggest that the metabolites respired in the TCA cycle are imported from the host (Fig. 4b).

The genome and transcriptome of ‘*G. incantans*’ revealed a strong host dependence on both amino acid and nucleotide supply (Fig. 4b; see Supplementary Note 4 for details). In contrast, the transcription profile of ‘*G. incantans*’ suggested that it could supply its host with riboflavin (vitamin B_2_), an essential vitamin that cannot be synthesized by most metazoans. Our analyses of the transcriptomic data of *Trichoplax* H2, as well as the genome and proteome of the closely related haplotype H1^24,36^, revealed that both seem to lack the known genes for synthesizing riboflavin (Supplementary Fig. 9) and rely on an external source of riboflavin (Supplementary Table 4). This suggests that when riboflavin availability is limiting for the host, ‘*G. incantans*’ could supplement the nutrition of its host.

‘*G. incantans*’ does not seem to be detrimental to *Trichoplax* H2, despite the fact that it has to import most of the compounds it needs for generating energy and biomass from its host. Our metagenomic, FISH and TEM data revealed 2−20 symbiont cells per fibre cell, so that the total number of ‘*G. incantans*’ cells per host individual is roughly the same as the number of host cells (Supplementary Note 5). This indicates closely regulated control of symbiont growth by the symbiont, the host or both partners. Pathogen abundances are typically orders of magnitude higher per host cell and often result in rapid exploitation and destruction of host cells and the impairment of host reproduction^37^. The relatively low abundance of ‘*G. incantans*’ in *Trichoplax* H2 together with the rapid doubling rates of these hosts (2−3 d in our aquaria) are in contrast to virulent pathogenic infections. Unlike all other known energy parasites, ‘*G. incantans*’ seems to generate its own ATP and might even modulate its host immune response to prevent apoptosis (Supplementary Note 4).

Bacterial phylotypes highly similar or identical to ‘*G. incantans*’ occur worldwide in aquatic environments. To assess how widespread the two *Trichoplax* symbionts are in other environments and hosts, we surveyed the ~300,000 publicly available amplicon-based 16S rRNA sequence libraries using the IMNGS pipeline. We did not find any sequences related to ‘*R. eludens*’, using a cut-off of 99% identity. In contrast, sequences highly similar or identical to ‘*G. incantans*’ were present in aquatic environments, both marine and limnic, from across the globe (Supplementary Table 7). Of the 8,026 libraries from aquatic environments, we found sequences that were at least 99% identical to ‘*G. incantans*’ in almost 10% of these libraries (*n* = 845). One third of the sequences were identical to ‘*G. incantans*’ and almost all were attributed to the genus *Grellia* on the basis of evolutionary placement analysis (Supplementary Fig. 10). This is remarkable for Midichloriaceae, because all other genera are much rarer and were found in only 0–55 libraries, depending on the genus (Supplementary Table 7). The presence of *Grellia* phylotypes in such a wide range of environments, including limnic ones, indicates that these bacteria have host ranges beyond placozoans. Indeed, our phylogenetic 16S rRNA analyses showed that sequences that group with the genus *Grellia* have been found in marine protists (*Eutreptiella*), sea cucumbers (*Apostichopus*) and oysters (*Crassostrea*), as well as in the limnic cnidarian *Hydra oligactis* (see Fig. 1a). The *Hydra* sequences came from specimens collected freshly from their natural environments and animals reared in the laboratory for more than 30 yrs, indicating the stability of this association in these hosts^38^.

The recent realization that human pathogens such as Chlamydiae, Legionellales and Rickettsiales have close relatives that live in hosts ranging from protists to fish and from aquatic and soil habitats has led to a paradigm shift in our view of the ecology and evolution of intracellular bacteria^27,39,40^. ‘*G. incantans*’ extends our conceptual understanding of the pervasiveness of such bacteria and shows that a single environmental rickettsial genus occurs worldwide in marine and limnic habitats. This remarkable distribution raises the question of whether all *Grellia* are host-associated. If ‘*G. incantans*’ had a free-living stage, this would be in contrast to all other known Rickettsiales that infect animals^27^.

Unlike other animals at the base of the animal tree, such as sponges, cnidarians or ctenophores, Placozoa is the only phylum in which intracellular bacteria have been observed in all individuals and haplotypes investigated. Intracellular symbiosis thus seems to be an invariant trait across this phylum. Our study identifies these bacteria in *Trichoplax* H2, shows that they are found in every specimen examined and defines the specificity and fidelity to the host cell type in which the symbionts reside.

How might the *Trichoplax* symbionts be transmitted within a growing individual and to its offspring? Within a host individual, the symbiont-containing cells could pass on their bacteria during division, or the symbionts could continuously reinfect host cells derived from aposymbiotic cells. However, little is known about cell turnover and proliferation in placozoans and it remains to be determined whether they even have stem cells. Similarly, we can only speculate on transmission during asexual reproduction (the main mode of reproduction in placozoans). In *Trichoplax* H2, which has been reproducing asexually in our aquaria for several years, the symbionts are transmitted with high fidelity, as all host individuals had both symbionts. Information on sexual reproduction, which is much rarer and has not been observed in nature, is too limited to allow us to know whether the symbionts are incorporated into resulting embryos. If not, the symbionts must be obtained from the environment. Symbiont uptake from the environment could explain why the midichloriacean symbionts of *Trichoplax* H1 and H2 do not belong to the same clade, although their hosts are very closely related and separated only a few decades ago^24^. This split could have been caused by their midichloriacean symbionts, as Rickettsiales are well known to induce reproductive incompatibility in insects^41^. Future studies of the microbiomes of the large number of extant haplotypes are needed to fully understand the ecology and evolution of symbioses between placozoans and their bacterial symbionts.

## Methods

### Isolation and cultivation

The placozoans were isolated from a coral tank at the Kewalo Marine Laboratory, University of Hawai’i at Mānoa in October 2015 by placing glass slides, mounted in plastic slide boxes that had the top and bottom cut out, into the tank for 10 d (ref. ^17^). Placozoans were identified under a dissection microscope, transferred to 400 ml glass beakers with 34.5‰ artificial seawater and fed weekly with 2 × 10^6^ cells ml^−1^ of *Isochrysis galbana* from a log-phase culture. Doubling times were 2−3 d at 25 °C in 34.5‰ artificial seawater and with a 16:8 h light:dark regime.

### Nucleic acid extractions

DNA was extracted from two single individuals from the *Trichoplax* H2 cultures using the DNeasy Blood & Tissue Kit (Qiagen) and DNA and RNA from three additional single individuals were extracted using the AllPrep DNA/RNA Micro Kit (Qiagen), according to the manufacturer’s protocols for both kits except for the following modifications. Proteinase K digests were performed overnight. Elution volumes were halved and all samples were eluted twice, reusing the first eluate. Elutions were carried out with a 10-min-long waiting step before centrifugation.

### DNA and RNA sequencing

Illumina-library preparation and sequencing were performed by the Max Planck Genome Centre. In brief, DNA/RNA quality was assessed with the Agilent 2100 Bioanalyzer (Agilent) and genomic DNA was fragmented to an average fragment size of 500 base pairs (bp). For the DNA samples, the concentration was increased (MinElute PCR Purification Kit; Qiagen) and an Illumina-compatible library was prepared using the Ovation Ultralow Library Systems Kit (NuGEN) according to the manufacturer’s protocol. For the RNA samples, the Ovation RNA-seq System V2 (NuGen) was used to synthesize complementary DNA and sequencing libraries were then generated with the DNA Library Prep Kit for Illumina (BioLABS). All libraries were size selected by agarose gel electrophoresis and the recovered fragments quality-assessed and quantified by fluorometry. For each DNA library, 14–22 million 150 bp paired-end reads were sequenced on a HiSeq 4000 (Illumina) and, for the RNA libraries, 150 bp single-end reads were sequenced to a depth of 42–44 million.

### Host mitochondrial 16S rRNA gene phylogenetic analyses

The metagenomic assembly was screened for the contig containing the host mitochondrial 16S rRNA gene (*m16S*) using BLAST v2.7.1 as implemented in Geneious R11^42^. The gene was extracted from the contig and aligned together with a database of publicly available *m16S* sequences using MAFFT v7.394 in G-Insi mode. The phylogenetic tree was reconstructed using FastTree v2.1.5 (ref. ^43^) with a GTR model, 20 rate categories and Gamma20 likelihood optimization, generating approximate likelihood-ratio-test values for node support. The tree was drawn with Geneious^42^. The tree was rooted with clade A placozoans^2^.

### Bacterial diversity 16S rRNA gene phylogenetic analyses

For the 16S rRNA gene database of all phylotypes recovered, the phyloFlash v3.0 beta1 pipeline (https://github.com/HRGV/phyloFlash) assembled full-length *SSU* genes for all samples. The dataset was aligned and phylogenetic trees were calculated and visualized as for the host *m16S* dataset above. The tree was rooted with the Eukarya and only the bacterial part of the tree is shown in this Letter.

### Genome analyses

Full-length 16S rRNA gene sequences were reconstructed for each metagenomic and metatranscriptomic library using phyloFlash v3.0 beta1 (https://github.com/HRGV/phyloFlash) from raw reads.

For assembly, adapters and low-quality reads were removed with bbduk v37.9 (https://sourceforge.net/projects/bbmap/) with a minimum quality value of 2 and a minimum length of 36; single reads were excluded from the analysis. Each library was error corrected using BayesHammer v3.62^44^. A combined assembly of all the libraries was performed using SPAdes 3.62 (ref. ^45^) with standard parameters and *k*-mers 21, 33, 55, 77 and 99.

The reads of each library were mapped back to the assembled scaffolds using bbmap v37.9 (https://sourceforge.net/projects/bbmap/) with the option fast = *t*. Scaffolds were binned on the basis of the mapped read data using MetaBAT v1.0^46^. The binning was refined using Bandage v0.8.1^47^ by collecting all contigs linked to the contig that contained the full-length 16S rRNA gene of the target organism. The bin quality metrics were computed with QUAST v5.0.2^48^ and the completeness for all bins was estimated using checkM v1.07 (ref. ^49^).

Annotation of the symbiont draft genomes was performed using RAST^50^ and verified with PSI-BLAST v2.7.1^51^ for selected genes discussed. Average nucleotide and amino acid identities between genomes^52,53^ were calculated with the ANI/AAI matrix calculator (http://enve-omics.ce.gatech.edu/g-matrix). Comparative analyses were conducted using the PATRIC database and services^54^. Pathway Tools v22.0^55^, in combination with the BioCyc database^56^, was used to analyse the metabolic capacities of ‘*G. incantans*’ and ‘*R. eludens*’. The genomes were screened for secretion systems and effectors using EffectiveDB^57^.

### Transcriptomic analyses

Adapters and rRNA gene reads were removed from the RNA-seq reads using bbduk v37.9. The gene expression for each symbiont genome bin and of the host (based on the published predicted proteome of *T.* *adhaerens* H1) was calculated from RNA-seq libraries using kallisto v0.45.0 with default settings^58^. Transcription levels were mapped onto metabolic pathways using Pathway Tools v22.0^55^.

### Proteomic analyses

Peptide samples for proteomics were prepared and quantified from two samples of 10 *Trichoplax* each and one sample of 30 *Trichoplax* specimens, as described by Kleiner et al.^59^ and according to the filter-aided sample preparation protocol described by Wisniewski et al.^60^. In addition to minor modifications described in Hamann et al.^61^, we did not clear the lysate by centrifugation after boiling the sample in lysis buffer. Instead, as the sample size was extremely limited (10 *Trichoplax* specimens = 0.2 µl), we loaded the whole lysate onto the filter units used for the filter-aided sample preparation procedure. Centrifugation times before column washes with 100 μl UA (8 M urea in 0.1 M Tris/HCl pH 8.5) were halved as compared to Hamann et al.^61^. Peptides were not desalted. Peptide concentrations were determined with the Pierce Micro BCA assay (Thermo Fisher Scientific) following the manufacturer’s instructions.

All samples were analysed by one-dimensional LC−MS/MS as described in Kleiner et al.^59^ with the modification that a 75 cm analytical column was used. Briefly, the sample containing 30 specimens was measured in technical replicate, for the others the whole sample was used in one analysis. The peptide (0.8–3 μg) was loaded with an UltiMate 3000 RSLCnano Liquid Chromatograph (Thermo Fisher Scientific) in loading solvent A (2% acetonitrile, 0.05% trifluoroacetic acid) onto a 5 mm × 300 µm ID C18 Acclaim PepMap100 pre-column (Thermo Fisher Scientific). Elution and separation of peptides on the analytical column (75 cm × 75 µm analytical EASY-Spray column packed with PepMap RSLC C18, 2 µm material, Thermo Fisher Scientific; heated to 60 °C) was performed at a flow rate of 225 nl min^−1^ using a 460 min gradient going from 98% buffer A (0.1% formic acid) to 31% buffer B (0.1% formic acid, 80% acetonitrile) in 363 min, then to 50% B in 70 min, to 99% B in 1 min and ending with 99% B. The analytical column was connected to a Q Exactive Plus Hybrid Quadrupole-Orbitrap mass spectrometer (Thermo Fisher Scientific) via an Easy-Spray source. Eluting peptides were ionized via electrospray ionization. Carry-over was reduced by two wash runs (injection of 20 µl acetonitrile, 99% eluent B) between samples. Data acquisition in the Q Exactive Plus was performed as in Petersen et al.^62^.

A database containing protein sequences from the *Trichoplax* host as well as the two symbionts was used. Sequences of common laboratory contaminants were included by appending the cRAP protein sequence database (http://www.thegpm.org/crap/). The final database contained 13,801 protein sequences. Searches of the MS/MS spectra against this database were performed with the Sequest HT node in Proteome Discoverer v2.2.0.388 (Thermo Fisher Scientific) as in Petersen et al.^62^. For protein quantification, normalized spectral abundance factors^63^ were calculated per species and multiplied by 100, to give the relative protein abundance as a percentage.

### Phylogenetic and phylogenomic analyses

A 16S rRNA gene database for ‘*G. incantans*’ was constructed using the assembled 16S rRNA gene sequence from each metagenomic library, the 20 best BLAST^64^ hits in the nr database and all other sequences of described *Candidatus* taxa in the Midichloriaceae. We added the five type strains with the best BLAST hit score (five species of *Rickettsia*) as an outgroup. We also screened the trace reads from the *Trichoplax* H1 genome project for reads containing Midichloriaceae 16S rRNA gene fragments using BLAST v2.7.1^64^, assembled them in Geneious R9 (http://www.geneious.com)^42^ and added the resulting sequence to the database. A similar search for margulisbacterial 16S rRNA fragments yielded no hits.

The 16S rRNA gene dataset was aligned using MAFFT v7.394^65^ and the phylogenetic tree was calculated using FastTree v2.1.10^43^ with a GTR model for nucleotide substitution. The tree was drawn with Geneious R9^42^.

For ‘*G. incantans*’, the database of genomes for phylogenetic analysis was compiled from all available genomes from the Midichloriaceae as well as representatives for all genera of the Anaplasmataceae and Rickettsiaceae. We also screened the assembly of the *Trichoplax* H1 genome project for contigs that belong to Midichloriaceae contamination using BLAST v2.7.1^64^ with the ‘*G. incantans*’ genome as implemented in Geneious R9 (http://www.geneious.com)^42^. The identified set of contigs corresponded to the set found by Driscoll et al.^25^ and was added to the database. We similarly searched for sequences related to ‘*R. eludens*’ in the H1 genome project, but no hits were detected.

For genome-based alignments of the amino acids of 43 conserved phylogenetic marker genes, the tree workflow as implemented in CheckM v1.0.11 was used^49^. For *Ruthmannia*, the genome bin data were integrated into a taxonomically selected part of the alignment from Hug et al.^11^ that covered all Melainabacteria and Cyanobacteria, WOR-1 and RBX-1 (Margulisbacteria), as well as five short branching Firmicutes as an outgroup. The phylogenetic reconstructions of the concatenated alignments were calculated using FastTree v2.1.10 with the WAG model for amino acid substitutions and visualized and analysed using iTOL^66^.

### Tag-sequence data analysis

The 16S rRNA gene sequences from ‘*G. incantans*’, as well as representative sequences from all characterized midichloriacean *Candidatus* taxa were used as query sequences to search the global collection of the microbial tag-sequencing library. The search was carried out using the IMNGS service^67^ with a minimal alignment length of 200 bp and a minimal identity of 99%. Identified amplicon libraries were grouped according to their deposited metadata. For the top 10% of libraries with the highest number of sequences from ‘*G. incantans*’, the habitat type (limnic or marine) and geolocation were manually collected from the deposited metadata and related publications. The detected 16S rRNA reads were aligned to the Rickettsiales dataset using MAFFT—addfragments and the evolutionary placements in the tree were performed using raxml v8.2.12^68^.

### TEM

Live specimens were high-pressure frozen with a HPM 100 (Leica Microsystem) in 3 mm aluminium sample holders, using hexane as filler as needed. The samples were transferred onto frozen acetone containing 1% osmium tetroxide and processed using a very quick freeze-substitution method^69^. After reaching room temperature, the samples were washed three times with acetone and infiltrated using centrifugation, modified after McDonald^70^, in 2 ml tubes sequentially with 25%, 50%, 75% and 2 × 100% Agar Low Viscosity Resin (Agar Scientific). For this process, the samples were placed on top of the resin and centrifuged for 30 s with a benchtop centrifuge (Heathrow Scientific) at 2,000*g* for each step. After the second pure resin step, they were transferred into fresh resin in embedding moulds and polymerized at 60 °C for 12 h.

Ultrathin (70 nm) sections were cut with an Ultracut UC7 (Leica Microsystem) and mounted on formvar-coated slot grids (Agar Scientific). They were contrasted with 0.5% aqueous uranyl acetate (Science Services) for 20 min and with 2% Reynold’s lead citrate for 6 min before imaging them at 20–30 kV with a Quanta FEG 250 transmission electron microscope (FEI Company) equipped with a scanning TEM detector using the xT microscope control software v6.2.6.3123.

For electron tomography, 300 nm serial sections were placed on formvar-coated 2 × 1 mm^2^ slot grids and stained with uranyl acetate and lead citrate. 30 nm gold fiducials were applied on both sides of the slot grid. Dual-axis tilt series (±60°, step size 1°) were acquired with a FEI Tecnai F30 300 kV electron microscope equipped with an Axial Gatan US1000 CCD camera. SerialEM software was used for the automated acquisition of tomographic tilt series^71^. Alignment and reconstruction of the tilt series were carried out with IMOD v4.9^72^. The serial tomograms were aligned with TrakEM2 v1.0i^73^ in Fiji^74^ and visualization and segmentation were carried out using the software Amira 3D v6.5.

### FISH

We used ARB−SILVA database 128 (ref. ^75^) and the ARB PROBE_DESIGN tool (the ARB software package v6.0.6)^76^ to design two FISH probes for each symbiont that were specific to their 16S rRNA sequences (Supplementary Table 2). We confirmed the specificity of the probes by comparing their sequences to all available sequences in the ARB−SILVA 128 database and Ribosomal Database Project release 11.5 (ref. ^77^). The most specific probe for ‘*R. eludens*’ had two mismatches to first non-target hit sequences; the most specific probe for *G. incantans* also matched the six most closely related *Grellia* sequences; detailed results are presented in Supplementary Table 2.

Specimens were fixed on coverslips with 2% formaldehyde and 0.1% glutaraldehyde in 1.5× PIPES, HEPES, EGTA and MgCl_2_ (PHEM) buffers modified from Montanaro et al.^78^ at 4 °C for 12 h. After three washing steps in 1.5× PHEM buffer, the samples were stored in 70% ethanol until use. Samples were rehydrated in PBS and hybridization was performed according to Manz et al.^79^. Mono-labelled-, DOPE-^80^ or MIL-^81^ probes (Supplementary Table 2) at a concentration of 8.4 pmol µl^−1^ were diluted with hybridization buffer containing 35% formamide, 900 mM NaCl, 20 mM Tris/HCl and 0.01% SDS at a ratio of 15:1. Whole animals were incubated in 30 µl of the probe/hybridization buffer mix at 46 °C in 250 µl PCR tubes for 3−4 h, followed by a 30-min-long washing step in washing buffer containing 700 mM NaCl, 20 mM Tris/HCl, 5 mM EDTA and 0.1% SDS. After a 10-min-long washing step in PBS, the animals were stained with DAPI for 30 min, washed twice again in PBS and mounted on glass slides in Vectashield mounting medium.

To test the probes designed for this study, 30 clonal individuals of *Trichoplax* H2 were pooled, fixed as described above, homogenized by sonication and applied to a filter. The parts of the filter were then tested with different formamide concentrations and the optimal formamide concentration was determined.

Fluorescence images were taken with a Zeiss LSM 780 equipped with a GaAsP detector or an Airyscan detector and a Plan-Apochromat 63×/1.4 and a Plan-Apochromat 100×/1.46 oil immersion objective using the ZEN software (black edition, 64bits, v14.0.1.201; Carl Zeiss Microscopy GmbH).

### Reporting Summary

Further information on research design is available in the Nature Research Reporting Summary linked to this article.

## Supplementary information

Supplementary Information Supplementary Notes 1−6, Supplementary Figs. 1−10, legend for Supplementary Video 1, Supplementary Tables 1 and 2, Supplementary Dataset 1 and Supplementary References.

Reporting Summary

Supplementary Table 3 ‘*R. eludens*’ transcriptome.

Supplementary Table 4 *Trichoplax* H2 transcriptome analysis.

Supplementary Table 5 ‘*G. incantans*’ transcriptome.

Supplementary Table 6 ‘*G. incantans*’ proteome.

Supplementary Table 7 *Grellia* is the most widely distributed midichloracean genus. This table shows IMNGS results for the characterized genera of Midichloriacea. Environmental categories were filtered for categories with at least ten cumulative hits accross all taxa.

Supplementary Video 1 3D rendering of reconstructed ‘*G. incantans*’ in *Trichoplax* H2 fibre cell rER.

## Data Availability

The metagenomic and metatranscriptomic raw reads and assembled symbiont genomes are available in the European Nucleotide Archive under Study Accession Number PRJEB30343. The mass spectrometry metaproteomics data and protein sequence databases were deposited in the ProteomeXchange Consortium^82^ via the PRIDE partner repository with the dataset PXD012106. The TEM 3D reconstruction data were deposited in figshare; the aligned tomography slices used for the reconstruction shown in Fig. 4 are available at https://figshare.com/s/886b869a9ada0264ffb2 (ref. ^31^).

## Code availability

The script used for the assembly graph-based binning is available at https://github.com/HRGV/tools_and_scripts.
