# Supplementary material for: Two intracellular and cell type-specific bacterial symbionts in the placozoan Trichoplax H2
Source: Nat Microbiol. 2019 Jun 10;4(9):1465–74. doi: 10.1038/s41564-019-0475-9 (PMC6784892; doi:10.1038/s41564-019-0475-9)
Supplement: Supplementary file 1 — Supplementary Notes 1−6, Supplementary Figs. 1−10, legend for Supplementary Video 1, Supplementary Tables 1 and 2, Supplementary Dataset 1 and Supplementary References. [file 41564_2019_475_MOESM1_ESM.pdf]

nature microbiology

SUPPLEMENTARY INFORMATION

https://doi.org/10.1038/s41564-019-0475-9

In the format provided by the authors and unedited.

Two intracellular and cell type-specific bacterial symbionts in the placozoan *Trichoplax* H2

Harald R. Gruber-Vodicka[1,7*], Nikolaus Leisch[1,7], Manuel Kleiner[2], Tjorven Hinzke[3,4,5], Manuel Liebeke[1], Margaret McFall-Ngai[6], Michael G. Hadfield[6*] and Nicole Dubilier[1*]

[1]Max Planck Institute for Marine Microbiology, Bremen, Germany. [2]Department of Plant and Microbial Biology, North Carolina State University, Raleigh, NC, USA. [3]Department of Pharmaceutical Biotechnology, Institute of Pharmacy, University of Greifswald, Greifswald, Germany. [4]Institute of Marine Biotechnology, Greifswald, Germany. [5]Department of Geoscience, University of Calgary, Calgary, Alberta, Canada. [6]Kewalo Marine Laboratory, Pacific Biosciences Research Center, University of Hawaiʻi at Mānoa, Honolulu, HI, USA. [7]These authors contributed equally: Harald R. Gruber-Vodicka, Nikolaus Leisch. *e-mail: hgruber@mpi-bremen.de; hadfield@hawaii.edu; ndubilie@mpi-bremen.de

Supplementary Information to

Two intracellular and cell type-specific bacterial symbionts in the placozoan *Trichoplax* H2

Harald R. Gruber-Vodicka[1*$], Nikolaus Leisch[1$], Manuel Kleiner[2], Tjorven Hinzke[3,4,5], Manuel Liebeke[1], Margaret McFall-Ngai[6], Michael G. Hadfield[6*], Nicole Dubilier[1*]

[1]Max-Planck Institute for Marine Microbiology, Celsiusstrasse 1, 28359 Bremen, Germany

[2]Department of Plant and Microbial Biology, North Carolina State University, Raleigh 27695, North Carolina, USA

[3]Department of Pharmaceutical Biotechnology, University of Greifswald, Institute of Pharmacy, Greifswald D-17489, Germany

[4]Institute of Marine Biotechnology, Greifswald, Germany

[5]Department of Geoscience, University of Calgary, Calgary, 2500 University Drive Northwest, Alberta T2N 1N4, Canada

[6]Kewalo Marine Laboratory, Pacific Biosciences Research Center, University of Hawaiʻi at Mānoa, Honolulu, HI 96813, USA

[$]contributed equally

*Corresponding authors

Contents

Contains or links to

Supplementary Notes 1 – 6

Supplementary Figures 1 – 10

Supplementary Video 1

Supplementary Tables 1 – 2

Supplementary Tables 3 – 7 are provided as separate files

Supplementary Datasets

Supplementary references

Supplementary Note 1 - Description of *Cand*. Grellia incantans

We name the *Candidatus* genus Grellia in honor of Karl G. Grell who rediscovered the placozoans after they had been erroneously dismissed as larvae of hydromeduse[1,2]. The species epithet incantans (pres. part. of Latin, *incantare* – bewitching or enchanting) refers to the ability of these bacteria to associate with hosts across the globe and their presence in both limnic and marine environments, possibly using the same book of songs for their wide range of environmental interactions.

Cand. Grellia incantans (from here on G. incantans) was co-isolated from a marine aquarium at the Kewalo Marine Laboratories, Honolulu, Hawaii together with its host, *Trichoplax* haplotype H2. It was detected in *Trichoplax* H2 hosts using fluorescence *in situ* hybridization (FISH) with the probes listed in Supplementary Table 2. G. incantans has not been cultivated outside of the *Trichoplax* H2 host. The cells are gram-negative small and thin rods (max. length 1200 nm, avg. length 541±359 nm, max. width 326 nm, avg. width 251±33 nm, n=19 cells). The cells are embedded in the host rough endoplasmic reticulum that is densely lined with ribosomes.

The recovered high quality genome bin for G. incantans was 1.26 Mb with an average GC content of 30.0% and 1219 protein coding sequences. The genome bin had an estimated completeness of 98.9% based on 108 conserved bacterial marker genes and no detectable contamination. We used the genome of G. incantans to BLAST-search the *Trichoplax* H1 genome for related sequences, and assembled a partial genome that was highly similar to the set of rickettsial contigs found in the *Trichoplax* H1 genome by Driscoll *et al*.[3]. Compared to the full genome of G. incantans, the 181 coding sequences recovered for the *Trichoplax* H1 phylotype (RETA1) had an average amino acid identity (AAI) of 64.38 % and had an average of 0.16 substitutions per site across 43 highly conserved genes. The AAI value is below the threshold of 65% AAI for genomic similarity within genera. In a similar manner, the 16S rRNA sequence of G. incantans was 98.3% identical to the one from RETA1 and clustered in a separate and well supported clade (Figure 1a). Based on the 16S rRNA tree

topology, as well as amino acid sequence identity, these two symbionts belong to two separate genera (Figure 1a).

Supplementary Note 2 - Description of *Cand*. Ruthmannia eludens

The *Candidatus* genus name Ruthmannia was named after August Ruthmann who devoted a significant part of his career to research on placozoans. The *Candidatus* species epithet eludens (pres. part. of Latin, *eludo* – escaping or fooling) refers to how well this symbiont has escaped detection compared to all other intracellular symbionts in metazoans, and how difficult the interpretation of a large part of its genome is, as there are no characterized homologs.

Cand. Ruthmannia eludens (from here on called R. eludens) was recovered from a marine aquarium at the Kewalo Marine Laboratories, Honolulu, Hawaii together with its host, *Trichoplax* H2. R. eludens was detected in *Trichoplax* H2 hosts using FISH with the probes listed in Supplementary Table 2. R. eludens has not been cultivated outside of the *Trichoplax* H2 host. The cells are Gram-negative rods (max. length 1158 nm, avg. length 669±233 nm, max. width 469 nm, avg. width 373±46 nm, n=37 cells).

We assembled a high quality 1.5 Mb metagenomic bin for R. eludens with an average GC content of 37.2% and 1356 protein coding sequences. Despite the small genome size, the genome draft had a computed completeness of 98.3% based on 108 conserved bacterial marker genes and no detectable contamination. Phylogenomic analysis based on 43 conserved marker genes placed R. eludens in the Margulisbacteria. Three classes of Margulisbacteria are currently characterized in the Genome Taxonomy Database (GTDB: http://gtdb.ecogenomic.org), WOR-1, GWF2-35-9, and ZB3 (Marinamargulisbacteria)[4,5], while a fourth class is known from termites (Termititenax)[6]. R. eludens grouped in the Marinamargulisbacteria, distantly related to single-cell amplified genomes and metagenome-assembled genomes from marine pelagic samples[4] (Figure 1c).

Based on transmission electron microscopy (TEM) data, R. eludens cells had a conspicuous, undulated outer membrane. The symbionts were in a host vacuole with as many as 15 symbionts co-occurring within a single host vacuole on a single cross-section (Figs. 3e, 3f and Supplementary Figure 7). The cells appeared to be anchored to the host membrane with fimbriae. These fimbriae-like structures may be products of a sec-dependent chaperone-usher (CU) gene set. CU systems are widespread in gram-negative bacteria, and encode essential proteins for the assembly and secretion of adhesive structures[7]. The CU system of R. eludens had remote homologs (25 - 30% amino acid identity) to those of bacteriovorous Deltaproteobacteria (Bdellovibrionales), which use their fimbriae to adhere to their bacterial prey[8]. Both chaperone and usher (PapC and PapD) were expressed, albeit at low levels (Supplementary Table 3).

Supplementary Note 3 - Ruthmannia eludens physiology

Transport across membranes

The only importers that could be annotated were for two non-essential amino acids (alanine and glutamate) and for cations important for metalloproteins (Ca^{2+}, Mg^{2+},Co^{2+}, Ni^{2+}, Mn^{2+}, Zn^{2+} and Fe^{3+}). Importers for alanine, Fe^{3+}, and Mn^{2+} were expressed, suggesting that the intracellular R. eludens, to some degree, rely on their host for acquiring nitrogen, iron and manganese.

To interact with the host cell, R. eludens must be able to export signaling molecules. Among the top ten most expressed genes was an outer membrane barrel protein that might serve as a porin, but its substrates could not be resolved, as for many other exporters. The substrate could only be identified for a single exporter gene, the cobalt efflux protein *corC*: Together with the anaerobic cobalt chaperone *cbiX*, *corC* could confer cobalt resistance, but this exporter was not expressed. The expressed SecAYG and YidCD proteins likely form an inner membrane protein assembly system that can translocate proteins from the cytoplasm to the periplasm, but none of the canonical secretion systems to export proteins across the outer membrane and into host cells were present in the genome.

Supplementary Note 4 - Grellia incantans physiology

As in the genomes of the tick and amoeba midichloriaceans, the G. incantans genome contained a cbb3 cytochrome oxidase typical for microaerophilic organisms.

Nucleotide and amino acid synthesis

The genome of G. incantans encoded only a subset of the genes for the *de novo* synthesis of nucleotides. Genes of this subset of the nucleoside/nucleotide biosynthesis as well as genes for parts of the nucleotide conversion pathways were detected in the transcriptome. Similarly, only a subset of the genes for amino acid synthesis were found, none of which were expressed (Figure 5b). The apparent lack of amino acid synthesis pathways could be compensated by a set of 18 importers for amino acids, many of which were expressed. Furthermore, we detected several importers for nucleotides, phosphorus and trace elements in the genome (Figure 5b).

Flagellar apparatus

A set of 35 genes encoding a fully functional flagellum, including all cytoplasmic, cell envelope-anchored and extracellular structural proteins and the necessary regulatory factors, was present in the genome. We never observed flagella in our electron microscopic analyses of nine host individuals. The presence of transcripts from genes for the cytoplasmic and cell-envelope anchor of the flagellum and a high expression of the filament-protein flagellin indicates that at least a part of the G. incantans population assembles or maintains flagella. This corresponds to the observed expression of flagella in other Rickettsiales during their intracellular stage[9]. A homologous set of flagellar genes was also found in the much larger genome of *Cand*. Jidaibacter, and was interpreted as an ancient feature of this bacterium[10]. In contrast, in *Cand*. Midichloria the set of flagellar genes is incomplete, and the flagellum likely is no longer functional[10]. Three genes of the flagellar apparatus were also detected in the partial rickettsial genome from the *Trichoplax* haplotype H1 genome (RETA1)[3]. As G. incantans and RETA1 belong to the sister clades of *Cand*. Jidaibacter and *Cand*. Midichloria (Figure 1a), it is parsimonious to assume that the last common ancestor (LCA) of the Midichloriaceae had a functional flagellum and was motile. Indeed, ultrastructural imaging and

genomic evidence across all families of Rickettsiales suggests that this was the case for the LCA of all Rickettsiales[11].

Transport across membranes

G. incantans most likely imports most amino acids, including all that are essential to the *Trichoplax* host, as full amino acid synthesis pathways were only present for aspartate, asparagine, glutamate and glutamine. We identified importers for at least ten amino acids including the essential amino acids methionine and lysine, of which two, for methionine and glutamate, were highly expressed. Typical for intracellular Rickettsia, the genome featured a large array of other importers, e.g. for nucleotides, co-factors like pantothenate (vitamin B5) and S-adenosylmethionine (AdoMet) or inorganics (magnesium, sulfate, iron). In contrast to the versatile importers, the G. incantans genome encoded only five exporters, a copper ABC-transporter, three multidrug exporters and a lysine exporter, but we did not find transcripts for any of these genes. The predicted import of nucleotides and amino acids as well as most other essential metabolites for energy generation and cell homeostasis indicates that G. incantans is dependent on its hosts for biomass formation.

Protective polyamines

In the Rickettsiaceae, millimolar concentrations of the polyamines putrescine and spermidine that are essential for DNA stabilization and protein biosynthesis, have been observed[12]. The expression of the polyamine importer PotABCD in G. incantans indicates that these protective molecules are also important in Midichloriaceae and likely constitute a conserved trait across Rickettsiales.

Antioxidants

Compared to *Cand*. Jidaibacter and *Cand*. Midichloria*,* which appear to have a very limited distribution, one of the factors that contributes to Grellia's wide habitat range could be that it has two variants of the potent antioxidant alkyl hydroperoxide reductase[13]. Similar to the multiple variants in *Bacillus subtilis*, these two variants could have different substrate specificities or expression patterns that would make G. incantans more tolerant against oxidative stress than other Midichloriaceae[14,15].

Type IV secretion systems effectors and the mitochondria of fiber cells

Rickettsiales are known to manipulate their hosts' cellular biology and evade recognition by its immune system[16]. These manipulations often rely on secretion systems and their secreted effectors. G. incantans encoded two variants of the type IV secretion system (T4SS). The T4SSs are versatile export systems that secrete proteins with a specific C-terminal peptide signature[17]. We detected 96 proteins with predicted T4SS specific C-terminal peptide signatures in the genome of R. incantans, several of which were among the most highly expressed genes. However, many had little homology to well characterized proteins and could therefore not be properly annotated. The three genes with the highest average expression that could be functionally annotated and had a predicted T4SS export-peptide signature may be involved in preventing apoptosis. Apoptosis is one of the most common responses of eukaryote cells to bacterial infection[18], and many pathogenic intracellular bacteria inhibit apoptosis by injecting effector proteins into their hosts through secretion systems. The three annotated genes were LSU ribosomal protein L7/L12, SSU ribosomal protein S11p and the chaperone protein DnaK. While L7/L12 and S11p could not be detected in all three transcriptomes, the highest consistently expressed and annotated protein with a predicted T4SS signature was DnaK, the bacterial homologue to heat shock protein 70 in eukaryotes (Hsp70/Hsp72). Eukaryotic Hsp70 prevents initiation of apoptosis in eukaryotic cells by blocking caspase-9 recruitment to the Apaf-1 apoptosome[18,19]. Eukaryotic Hsp72 has been shown to dampen the unfolded protein response of the rER, a cellular rescue mechanism that is tightly linked to the detection of viral or bacterial interference with eukaryotic protein expression[20]. G. incantans may export DnaK to exploit these two mechanisms and downregulate an immune response of the host. A similar use of DnaK was reported for the alphaproteobacterial pathogen *Brucella*[21].

The mitochondria in all fiber cells have an enlarged morphology and are alternatingly stacked with vesicles, forming so-called mitochondrial complexes that are a key feature of placozoan fiber cells[2]. G. incantans is specific to the fiber cells and likely present in all of these cells, but we could not find genomic evidence that it is involved in the formation of the aberrant mitochondria, despite the fact

that two effectors with mitochondrial signal peptides are highly expressed. The first predicted effector is a conjugative plasmid relaxase associated to the transfer of DNA via the type IV secretion system and unlikely to affect mitochondrial morphology. The second is a small protein (134 amino acids) with no characterized homologues that is conserved across Midichloriaceae. As neither *Cand*. Jidaibacter nor *Cand*. Midichloria induce mitochondrial complexes in their host cells, its function remains to be shown.

Supplementary Note 5 - Metagenomics based symbiont cell number estimates

We estimated the number of G. incantans cells per host cell using metagenomic coverages as proxies for cell abundances of the symbionts and *Trichoplax*. The read coverage in metagenomes is directly related to copy numbers of chromosomes and therefore to cell numbers. The coverage for both symbiont chromosomes in the five metagenomes was variable, but as low or lower than for the host nuclear genome, down to more than 10x lower. An average *Trichoplax adhaerens* individual was estimated to consist of approximately 50.000 host cells [22] and we estimated similar cell numbers for the morphologically indistinguishable H2 haplotype. Using these estimates, we translated the metagenomics coverage ratios to cell counts of 5.000 - 50.000 intracellular bacteria for each of the two symbionts. G. incantans resides in the fiber cells that extend throughout the whole animal as a connective cell layer[22] (Figure 3). Given that on average only 5% of the cells in a host individual are fiber cells[22], this corresponds to 2 - 20 symbiont cells per fiber cell.

Supplementary Note 6 – symbiont transmission and fidelity

Although intracellular symbionts are a shared characteristic of all placozoans investigated to date, only little is currently known about the diversity of these symbionts across the 19 cryptic species within this phylum. Our study provides the first insights into how these symbioses may have evolved in two very closely related *Trichoplax* haplotypes, H1 and H2. These two haplotypes putatively separated only decades ago[23]. Intriguingly, their symbioses appear to have followed very different

trajectories. While all *Trichoplax* H2 specimens we investigated in this study had the symbiont R. incantans of the Margulisbacteria, neither this symbiont nor any of its close relatives appears to be present in *Trichoplax* H1 (see Methods). These findings suggest that either: i) the last common ancestor of *Trichoplax* H1 and H2 had a margulisbacterial symbiont that was lost in the H1 lineage; or ii) the last common ancestor of these two host haplotypes did not have a margulisbacterial symbiont, and the H2 lineage acquired this symbiont recently, after separating from H1.

Similarly, the rickettsial symbionts of *Trichoplax* H1 and H2 may have also been acquired independently. The rickettsial symbionts of these two host lineages belong to two different bacterial genera and their 16S rRNA sequences differ by 1.7%. Rates of 16S rRNA divergence in bacteria are estimated to range between 2-11% per 100 million years[24]. Even if these estimates are off by one or even two orders of magnitude, the H1 and H2 symbionts are likely to have diverged from each other at least one million years ago. Assuming that the H1 and H2 hosts separated only decades ago, the vast difference in the time the hosts diverged compared to the divergence time of their symbionts implies that co-speciation could not have occurred. Instead, we envision the following scenarios: 1) The last common ancestor of H1 and H2 had a Grellia-related symbiont. In H1, the Grellia symbiont was replaced by a bacterium from the RETA1 clade, while H2 retained its Grellia symbiont. Or vice versa, H1 retained its symbiont, and H2 acquired a symbiont from the Grellia lineage. 2) The last common ancestor of H1 and H2 had a symbiont unrelated to the H1 and H2 symbionts. H1 then acquired a symbiont from the RETA1 lineage, while H2 acquired its symbiont from the Grellia genus.

Supplementary Figures

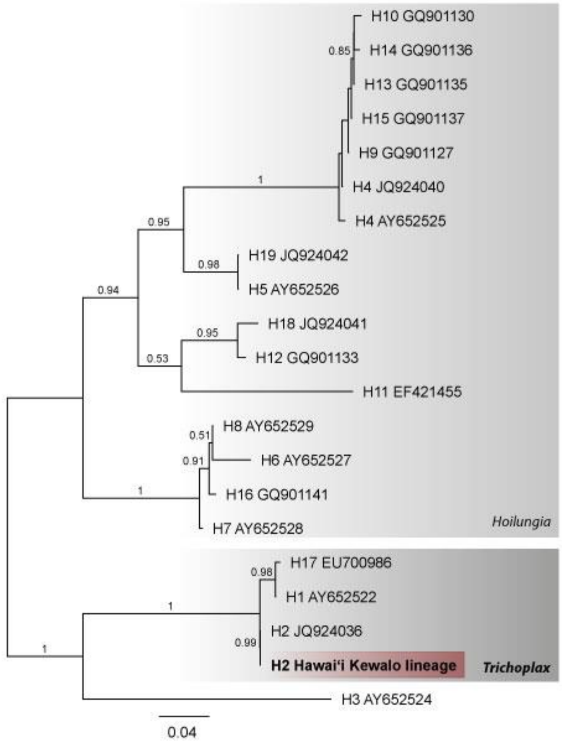

Supplementary Figure 1 – Based on the mitochondrial 16S rRNA, the Kewalo *Trichoplax* is haplotype H2

Phylogenetic tree based on the alignment of the mitochondrial large subunit rRNA (16S), support values below 0.5 are not shown. Scale bar indicates substitutions per site. The placozoan genera, haplotypes and the accession numbers are indicated.

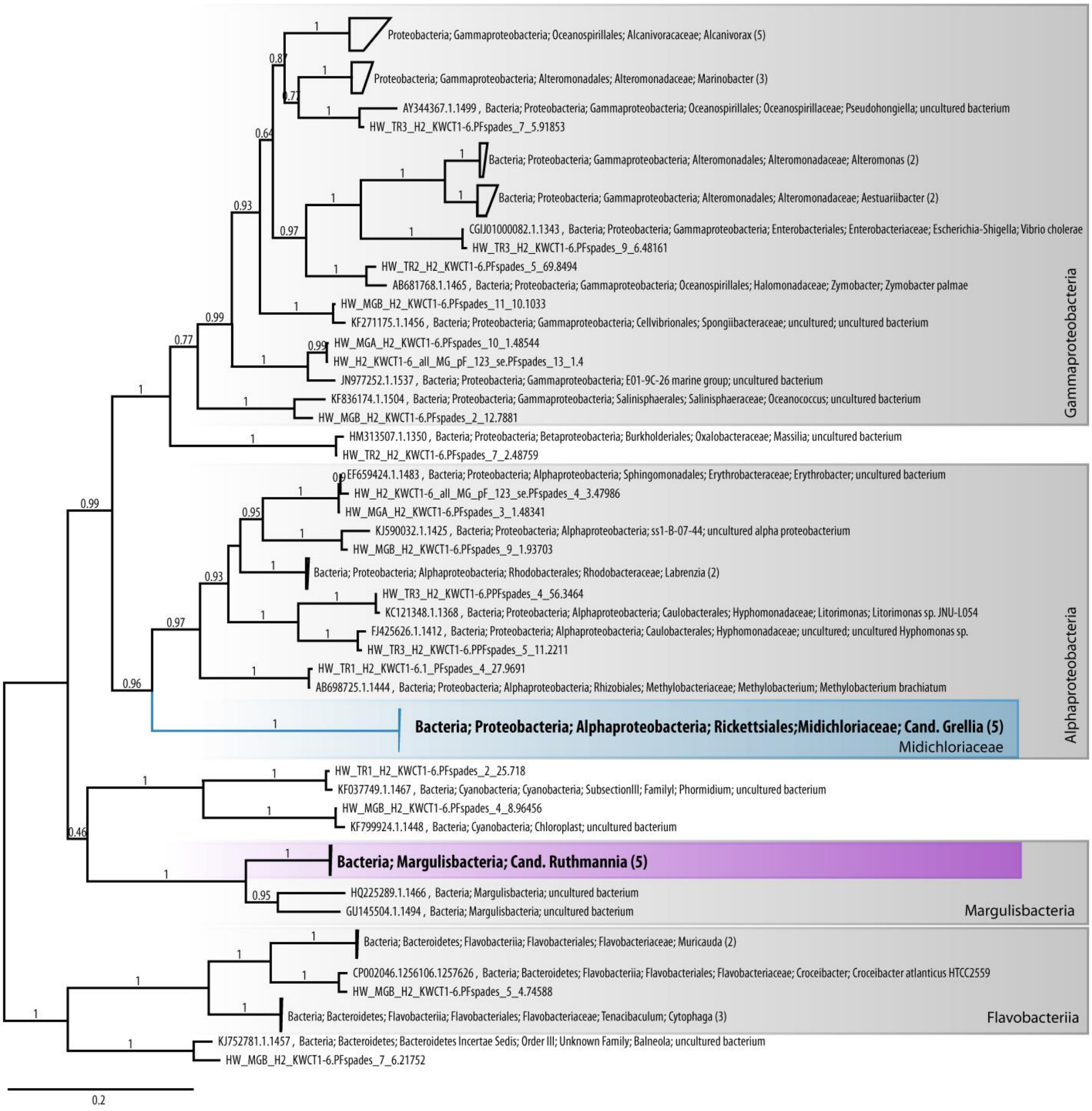

Supplementary Figure 2 – Full length 16S rRNA based diversity of bacteria associated with five single individuals of the Kewalo *Trichoplax* H2 lineage

Phylogenetic tree based on the alignment of the bacterial 16S rRNA gene. Scale bar indicates substitutions per site. Major bacterial lineages that were present in at least three samples are indicated in grey.

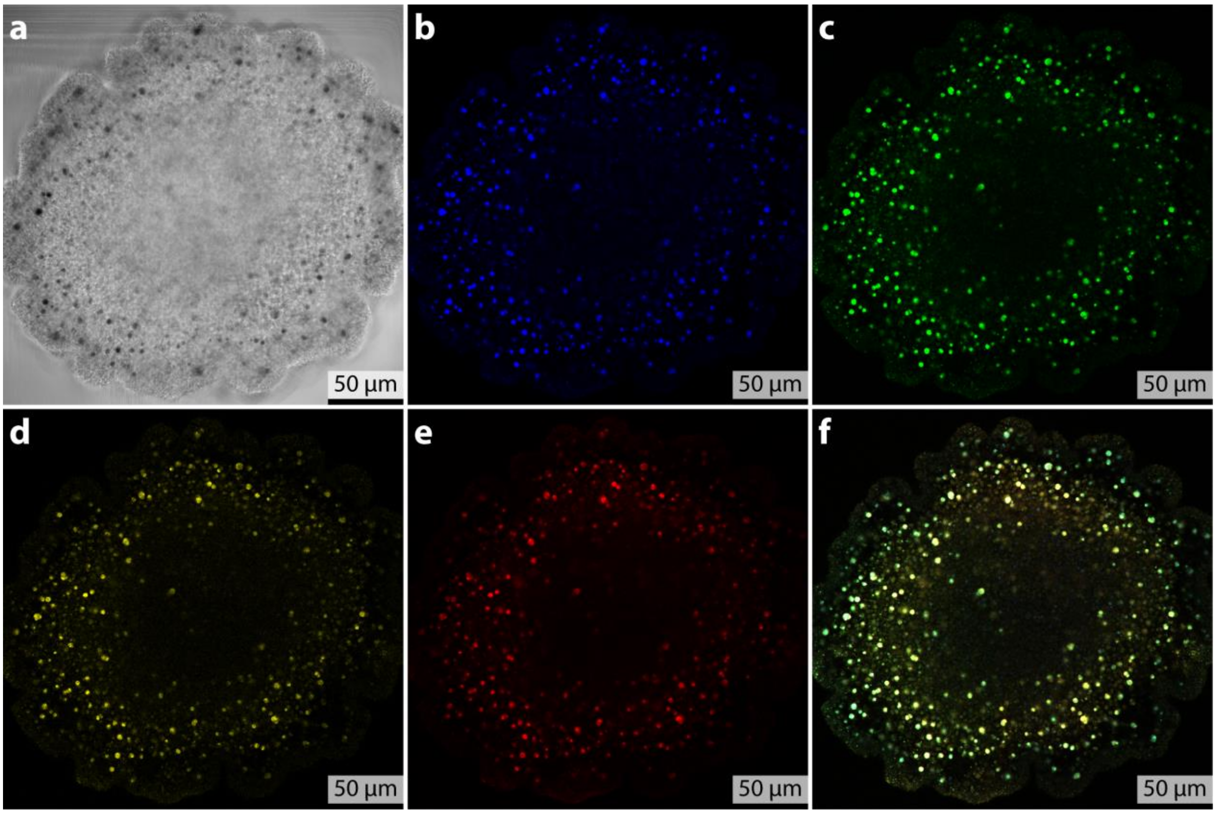

Supplementary Figure 3 – Autofluorescence of *Trichoplax* H2

All panels are the results from a single experiment. **a-f,** Images of the same whole mount *Trichoplax* H2 specimen **a,** Transmitted light image of the animal **b**-**e**, Autofluorescence induced by the most commonly used laser-lines of a confocal laser scanning microscope. **f**, Overlay of **b**-**e**

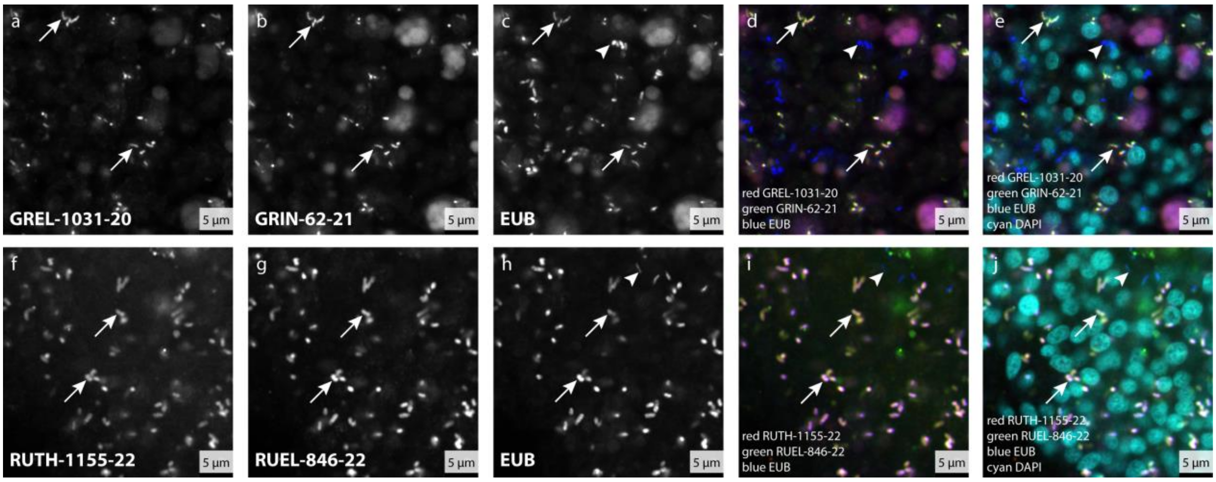

Supplementary Figure 4 - Fluorescence in-situ hybridization of the two *Trichoplax* H2 symbionts

a-j The results are representative of five independent experiments. **a** and **b,** Probes specific for G. incantans **c** and **h,** Eubacterial probe **f** and **g**, Probes specific for R. eludens; **d** and **i,** Overlay of the three probes shown in the images to the left**,** with DAPI added in **e** and **j**. In the top row, the white arrows point to G. incantans labeled with the two probes specific to this symbiont and the eubacterial probe. The white arrowhead shows R. eludens, which was only labeled by the EUB probe. In the bottom row, the white arrows point to R. eludens labeled with the two probes specific to this symbiont and the eubacterial probe. The white arrowhead shows G. incantans, which was only labeled by the EUB probe.

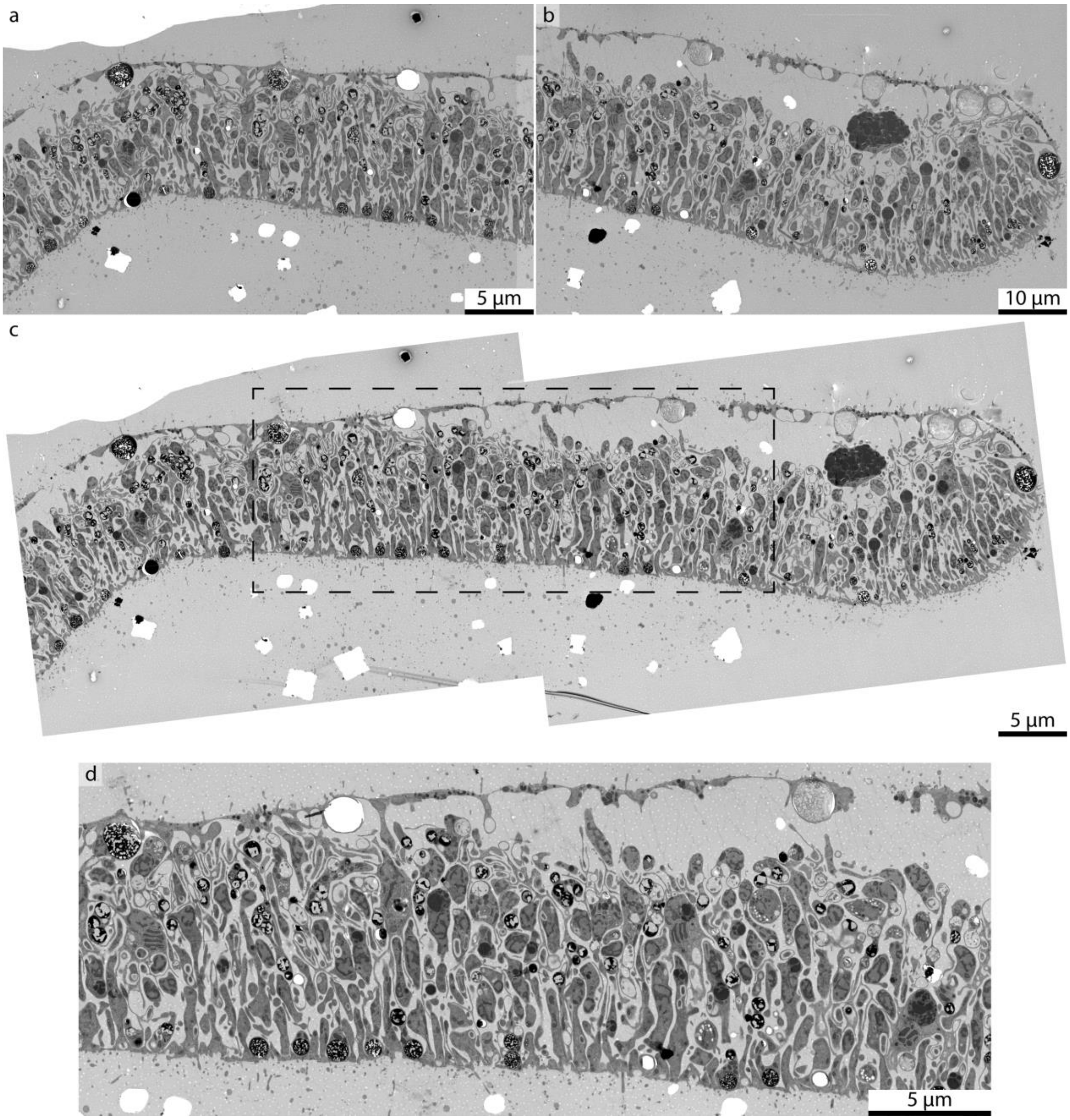

Supplementary Figure 5 – Transmission electron microscopic raw image data used for the false coloration shown in Figure 2b

a – d The results are representative of three independent experiments**. a** and **b,** The two original images **c,** The combined image **d,** Detail used in the main figure 2b (indicated with a dotted rectangle in **c**).

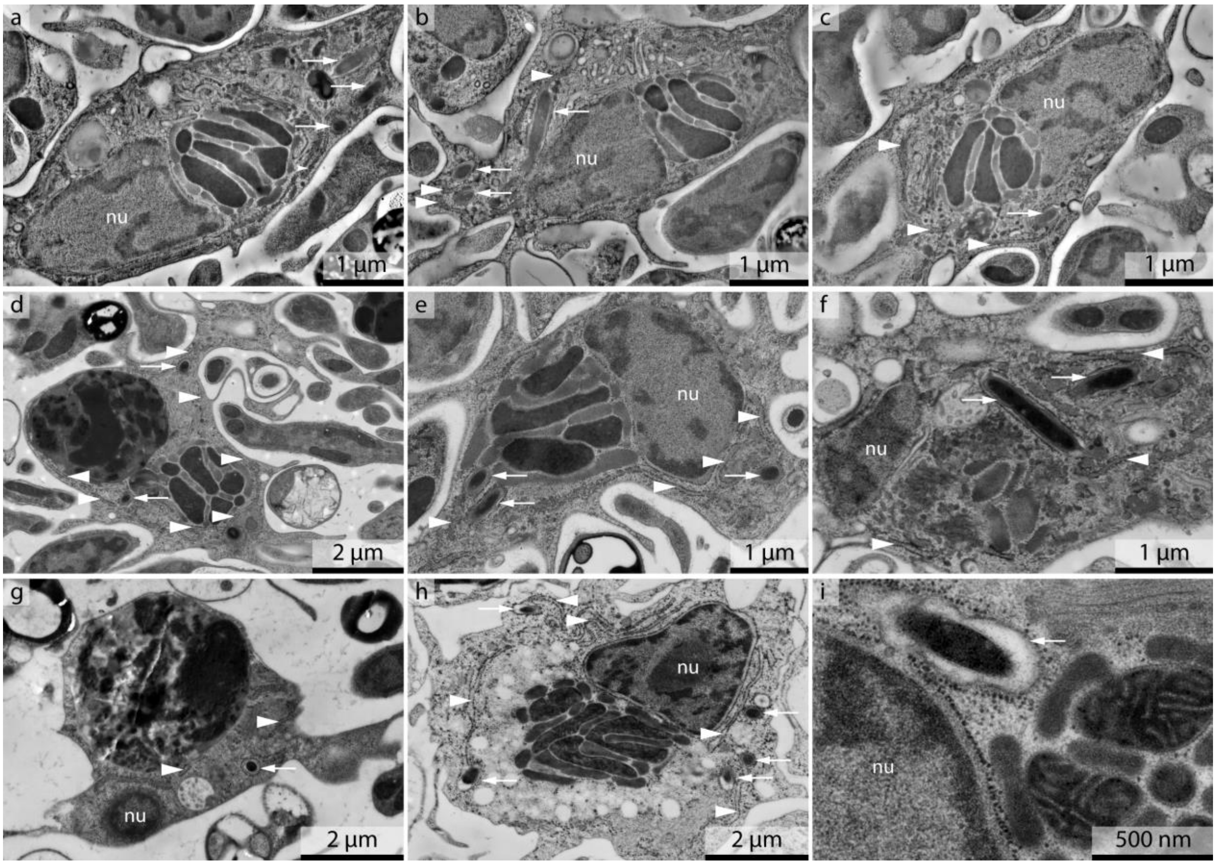

Supplementary Figure 6 – Transmission electron microscopy images of fiber cells and the localization of G. incantans.

a-**i** The results are representative of three independent experiments**.** **a**-**c,** Individual slices extracted from a tomogram **d**-**f,** Thin sections of epon embedded animals **g**-**i,** Thin sections of an LR-White embedded animal. nu indicates the nucleus, arrowheads point towards the rough endoplasmic reticulum and arrows point towards G. incantans located within the rER

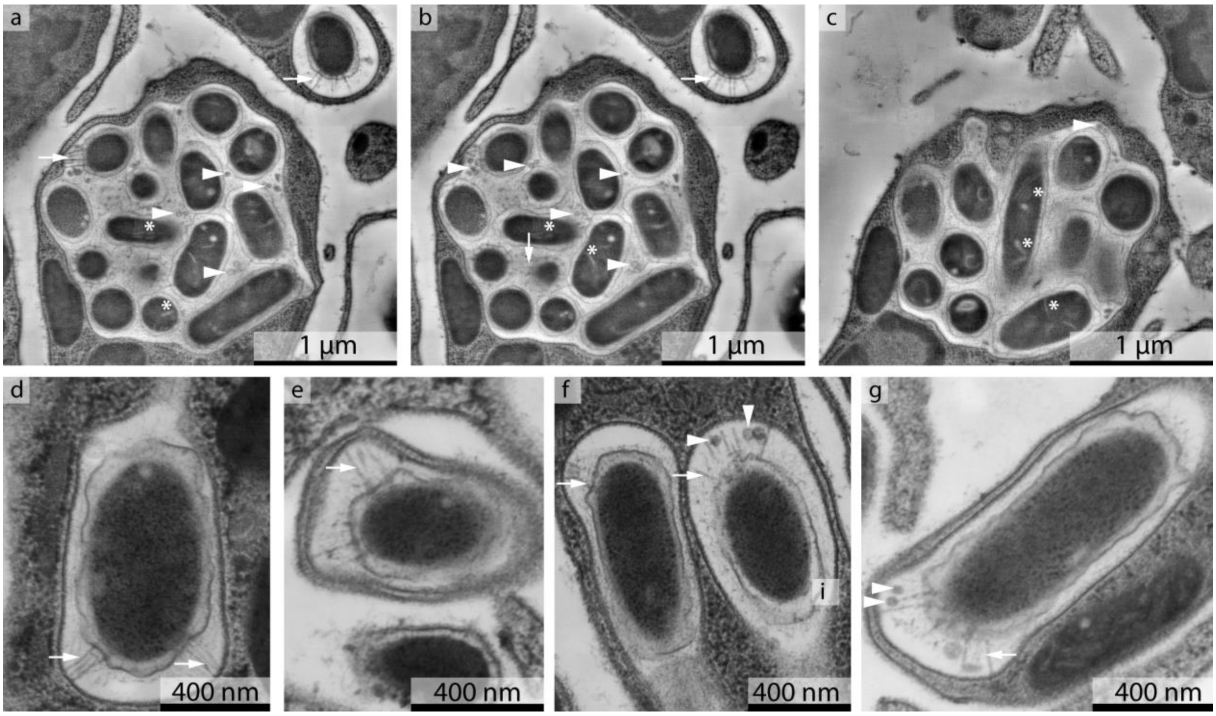

Supplementary Figure 7 – Transmission electron microscopy of ventral epithelial cells and localization of Ruthmannia eludens

a-g The results are representative of three independent experiments**. a-c,** Individual slices extracted from a tomogram showing R. eludens in the ventral epithelial cell **d-g,** R. eludens in higher magnification. Arrowheads point to outer membrane vesicles, arrows point towards the fimbriae like structures and the asterisk indicates internal structures in R. eludens.

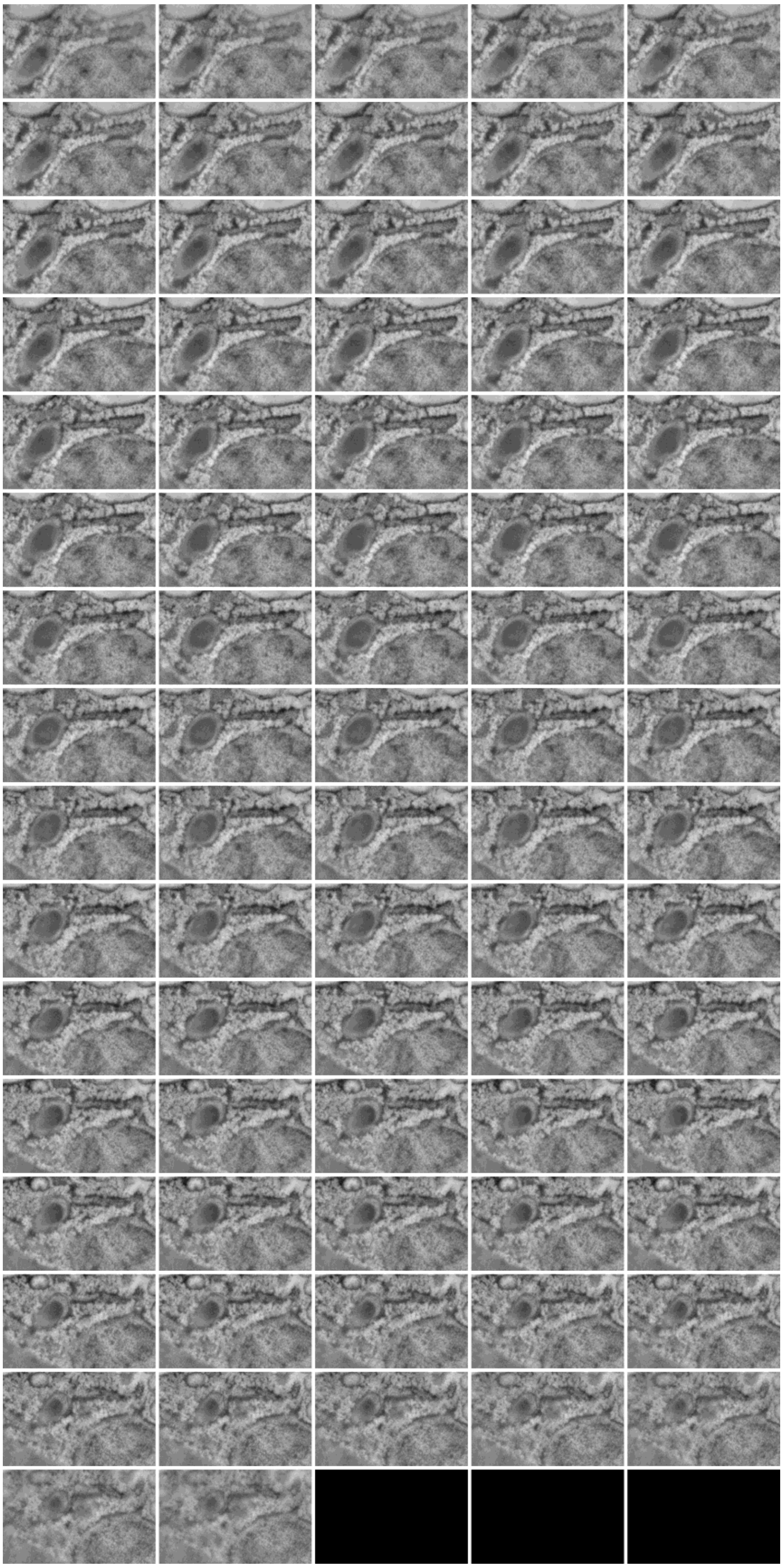

Supplementary Figure 8 - Electron tomography of Grellia incantans

The results are from a single experiment. Cropped detail from a single tomogram of a 300 nm section, showing G. incantans surrounded by rER, which in turn is connected to the nuclear membrane. Six individual slices of this tomogram were shown in Figure 4.

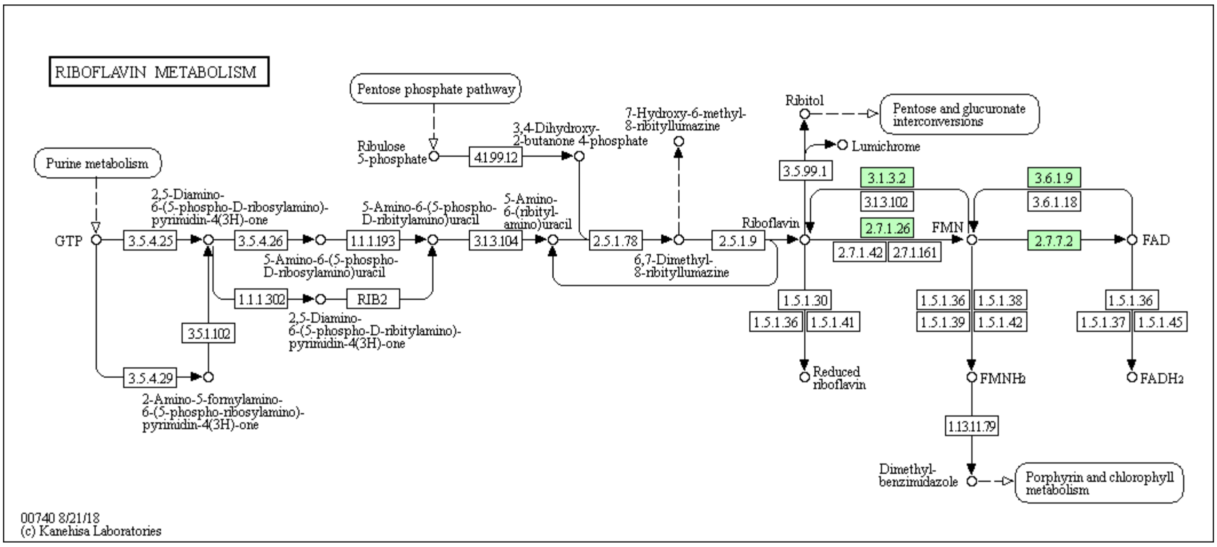

Supplementary Figure 9 - Riboflavin KEGG map of *Trichoplax* H1 genome

Genes present in the genome of *Trichoplax* H1 are shown in green

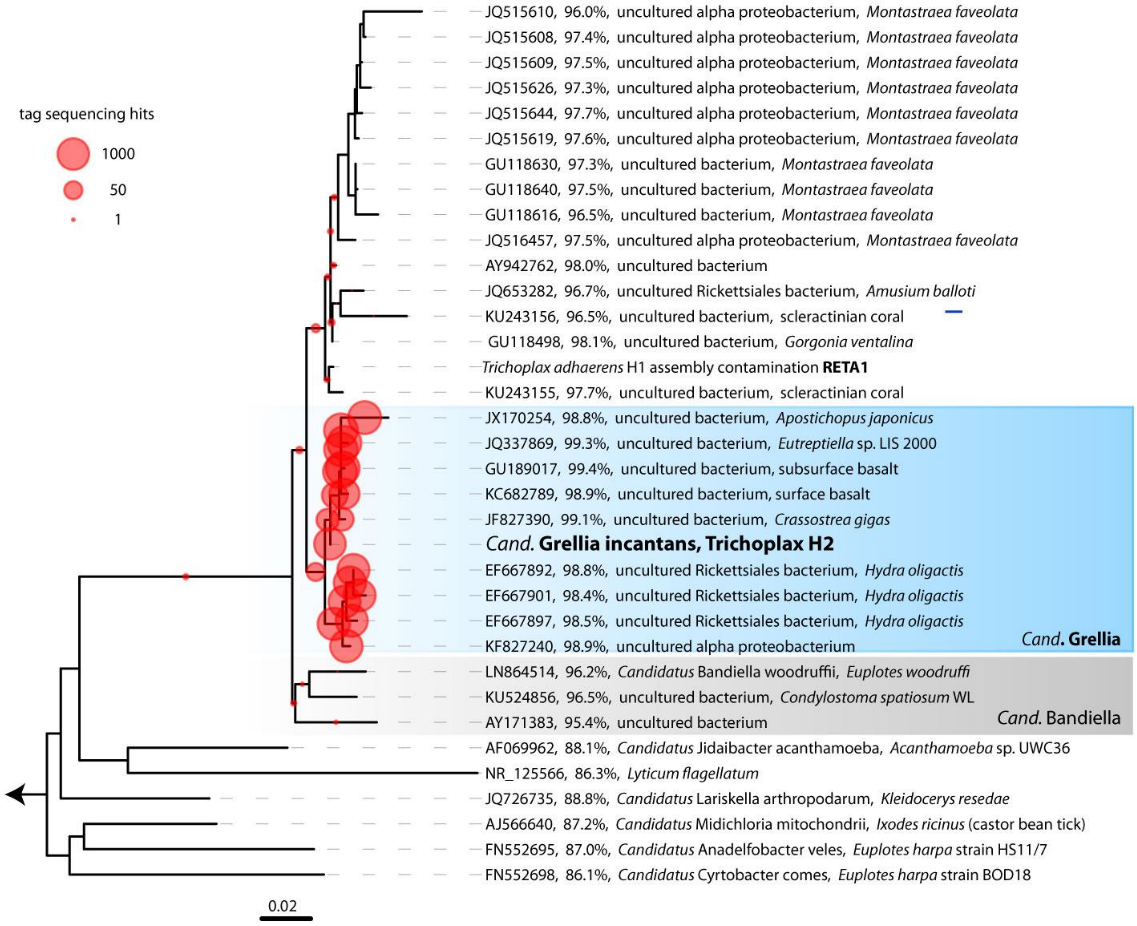

Supplementary Figure 10 – EPA of SRA sequences that were ≥ 99% identical to Grellia incantans in 16S rRNA gene tree of Midichloriaceae

Scale bar indicates substitutions per site. 16S rRNA tree of G. incantans and related Midichloriaceae; for each sequence, the accession number, the % identity to G. incantans, and the published taxonomic names and hosts, where available, are indicated. EPA placements of SRA sequences detected with IMNGS to be at least 99% identical to the G. incantans sequence are indicated in red circles, circle size indicates numbers of placements.

Supplementary Video

Supplementary Video 1 – 3D rendering of reconstructed G. incantans in *Trichoplax* H2 fiber cell rER.

G. incantans (light blue), rER (yellow) and the nucleus (red) of a fiber cell are superimposed on a virtual slice of the 3D TEM tomography stack.

Supplementary Tables

Supplementary Table 1 – The microbiome is dominated by Grellia incantans and Ruthmannia eludens.

Shown are SSU hit read counts in reads per million SSU reads that could be mapped to any of the taxa found with phyloFlash. Only taxa above 1000 SSU reads per million are shown. Colors indicate levels of abundance in continuous 3-color gradient (red – highest value, yellow – 50 percentile, green – lowest value).

Taxon	SSUpm TR1	SSUpm TR2	SSUpm TR3	SSUpm MGA	SSUpm MGB	AVERAGE
Trichoplax H2	978747	928343	941933	539873	258020	729383
Grellia incantans	8195	25212	19241	22190	10179	17003
Ruthmannia eludens	7959	19472	21559	5684	3923	11719
Alcanivorax	445	8331	2792	1347	19903	6564
Marine Gammas E01-9C-26	0	8	3	10084	8477	3715
unc. Alpha	3	6	75	2782	15440	3661
Marinobacter	4	93	227	2057	15386	3553
Alcanivorax	162	3541	3040	0	5916	2532
Erythrobacter	10	52	67	7750	4203	2416
Methylobacterium	771	4619	2495	350	1652	1977
Labrenzia	122	607	2668	0	3578	1395

Supplementary Table 2 - Overview of FISH probes used in this study

To overcome high autofluorescence of the host (see Supplementary Figure 3), a modified FISH protocol that used double and quadruple labeled probes was employed, see Fluorochrome column for details.

Name	Sequence (5'-->3')	Length	Formamide concentration	Fluorochrome	Reference	RDP hits - 0 mismatches	RDP hits - 1 mismatches	RDP hits - 2 mismatches
RUTH-1155-22	TTTCCACAGGCAGTCTCTTGTG	22	35%	Atto-647	This study	6 / 5x ZB3, 1x unclassified	18 / 10x ZB3, 7x unclassified, 1x Proteobacteria	48 / 30x ZB3, 15x unclassified, 2x Deinococcus-Thermus, 1x Proteobacteria
RUEL-846-22	ATTATCTGGGGCACTGAAAGGG	22	35%	Atto-594<br>4x Atto-594	This study	none	none	28 / 11x ZB3, 17x unclassified
GREL-1031-20	CTGTGATAGTCCAGCCGAAC	20	35%	Atto-647	This study	31/ 21 Proteobacteria, 3x Verrucomicrobia, 7x unclassified	195 / 111x Proteobacteria, 53x Verrucomicrobia, 24x Actinobacteria, 7x unclassified	1158 / 840x Proteobacteria, 213x Verrucomicrobia, 75x Actinobacteria, 24x unclassified, 3x Cyanobacteria, 1x Bacteroidetes, 1x Marinimicrobia, 1x Nitrospinae
GRIN-62-21	GCACAAATATCGTCCGTTCGA	21	35%	4xAtto-594<br>2xAtto-647	This study	6 / 6x Proteobacteria	17/ 17x Proteobacteria	30 / 23x Proteobacteria, 6x Firmicutes, 1x Actinobacteria
EUB I 338	GCTGCCTCCCGTAGGAGT	18	35%	Atto-488	[21]	-	-	-
EUB II 338	GCAGCCACCCGTAGGTGT	18	35%	Atto-488	[22]	-	-	-
EUB III 338	GCTGCCACCCGTAGGTGT	18	35%	Atto-488	[22]	-	-	-
NON 338	ACTCCTACGGGAGGCAGC	18	35%	Cy3	[23]	-	-	-

Supplementary Datasets

Supplementary Dataset 1 – Aligned tomography stack

The aligned tomography slices used for the reconstruction shown in Figure 4 are available at https://figshare.com/s/886b869a9ada0264ffb2 (doi 10.6084/m9.figshare.7429793).

Supplementary References

1 Krumbach, T. *Trichoplax*, die umgewandelte Planula einer Hydramedusae. *Zoologischer Anzeiger* **31**, 450-454 (1907).

2 Grell, K. G. & Benwitz, G. Die Ultrastruktur von *Trichoplax adhaerens* F.E. Schulze. *Cytobiologie* **4**, 216-240. (1971).

3 Driscoll, T., Gillespie, J. J., Nordberg, E. K., Azad, A. F. & Sobral, B. W. Bacterial DNA Sifted from the *Trichoplax adhaerens* (Animalia: Placozoa) Genome Project Reveals a Putative Rickettsial Endosymbiont. *Genome Biol. Evol.* **5**, 621-645, doi:10.1093/gbe/evt036 (2013).

4 Matheus Carnevali, P. B. *et al.* Hydrogen-based metabolism - An ancestral trait in lineages sibling to the Cyanobacteria. *bioRxiv*, doi:preprint at https://doi.org/10.1101/328856 (2018).

5 Parks, D. H. *et al.* A standardized bacterial taxonomy based on genome phylogeny substantially revises the tree of life. *Nat. Biotechnol.* **36**, 996-1004, doi:10.1038/nbt.4229 (2018).

6 Utami, Y. D. *et al.* Genome analyses of uncultured TG2/ZB3 bacteria in 'Margulisbacteria' specifically attached to ectosymbiotic spirochetes of protists in the termite gut. *ISME J.*, doi:10.1038/s41396-018-0297-4 (2018).

7 Busch, A. & Waksman, G. Chaperone–usher pathways: diversity and pilus assembly mechanism. *Philos. Trans. R. Soc. Lond., B, Biol. Sci.* **367**, 1112-1122, doi:10.1098/rstb.2011.0206 (2012).

8 Rendulic, S. *et al.* A Predator Unmasked: Life Cycle of *Bdellovibrio bacteriovorus* from a Genomic Perspective. *Science* **303**, 689, doi:10.1126/science.1093027 (2004).

9 Vannini, C. *et al.* Flagellar Movement in Two Bacteria of the Family Rickettsiaceae: A Re-Evaluation of Motility in an Evolutionary Perspective. *PloS one* **9**, e87718, doi:10.1371/journal.pone.0087718 (2014).

10 Schulz, F. *et al.* A *Rickettsiales* symbiont of amoebae with ancient features. *Environ. Microbiol.* **18**, 2326-2342, doi:10.1111/1462-2920.12881 (2016).

11 Castelli, M., Sassera, D. & Petroni, G. in *Rickettsiales: Biology, Molecular Biology, Epidemiology, and Vaccine Development* (ed Sunil Thomas) 59-91 (Springer International Publishing, 2016).

12 Speed, R. R. & Winkler, H. H. Acquisition of polyamines by the obligate intracytoplasmic bacterium *Rickettsia prowazekii*. *J. Bacteriol.* **172**, 5690-5696, doi:10.1128/jb.172.10.5690-5696.1990 (1990).

13 Ezraty, B., Gennaris, A., Barras, F. & Collet, J.-F. Oxidative stress, protein damage and repair in bacteria. *Nat. Rev. Microbiol.* **15**, 385-396, doi:10.1038/nrmicro.2017.26 (2017).

14 Broden, N. J. *et al.* Insights into the Function of a Second, Nonclassical Ahp Peroxidase, AhpA, in Oxidative Stress Resistance in *Bacillus subtilis*. *J. Bacteriol.* **198**, 1044-1057, doi:10.1128/jb.00679-15 (2016).

15 Zwick, J. V. *et al.* AhpA is a peroxidase expressed during biofilm formation in *Bacillus subtilis*. *MicrobiologyOpen* **6**, e00403-n/a, doi:10.1002/mbo3.403 (2017).

16 Walker, D. H. & Ismail, N. Emerging and re-emerging rickettsioses: endothelial cell infection and early disease events. *Nat. Rev. Microbiol.* **6**, 375, doi:10.1038/nrmicro1866 (2008).

17 McDermott, J. E. *et al.* Computational Prediction of Type III and IV Secreted Effectors in Gram-Negative Bacteria. *Infect. Immun.* **79**, 23-32, doi:10.1128/iai.00537-10 (2011).

18 Gross, A., Terraza, A., Ouahrani-Bettache, S., Liautard, J.-P. & Dornand, J. In Vitro *Brucella suis* Infection Prevents the Programmed Cell Death of Human Monocytic Cells. *Infect. Immun.* **68**, 342-351, doi:10.1128/IAI.68.1.342-351.2000 (2000).

19 Beere, H. M. *et al.* Heat-shock protein 70 inhibits apoptosis by preventing recruitment of procaspase-9 to the Apaf-1 apoptosome. *Nat. Cell Biol.* **2**, 469, doi:10.1038/35019501 (2000).

20 Gupta, S. *et al.* HSP72 Protects Cells from ER Stress-induced Apoptosis via Enhancement of IRE1α-XBP1 Signaling through a Physical Interaction. *PLoS Biol.* **8**, e1000410, doi:10.1371/journal.pbio.1000410 (2010).

21 Liu, N. *et al.* The Rab1 in host cells modulates *Brucella* intracellular survival and binds to *Brucella* DnaK protein. *Arch. Microbiol.* **198**, 923-931, doi:10.1007/s00203-016-1246-0 (2016).

22 Smith, C. L. *et al.* Novel cell types, neurosecretory cells, and body plan of the early-diverging metazoan *Trichoplax adhaerens*. *Curr. Biol.* **24**, 1565-1572, doi:10.1016/j.cub.2014.05.046 (2014).

23 Kamm, K., Osigus, H. J., Stadler, P. F., DeSalle, R. & Schierwater, B. *Trichoplax* genomes reveal profound admixture and suggest stable wild populations without bisexual reproduction. *Sci. Rep.* **8**, doi:10.1038/s41598-018-29400-y (2018).

24 Kuo, C.-H. & Ochman, H. Inferring clocks when lacking rocks: the variable rates of molecular evolution in bacteria. *Biol. Direct* **4**, 35, doi:10.1186/1745-6150-4-35 (2009).
